# Estimating on-farm genotypic performance and variability using ranking data

**DOI:** 10.1007/s00122-026-05319-1

**Published:** 2026-08-18

**Authors:** Hugo Dorado-Betancourt, Fred van Eeuwijk, Jacob van Etten, Julian Ramirez-Villegas, Kaûe de Sousa, Happy Daudi, Michael Ndegwa, Thiago Mendes, Joost van Heerwaarden

**Affiliations:** 1 Mathematical and Statistical Methods (Biometris), Wageningen University & Research, Wageningen, The Netherlands; 2 Plant Production Systems, Wageningen University & Research, Wageningen, The Netherlands; 3 Digital Inclusion, Bioversity International, Montpellier, France; 4 Climate Action, International Center for Tropical Agriculture (CIAT), Cali, Colombia; 5 Department of Agricultural Sciences, University of Inland Norway, Hamar, Norway; 6 Dryland Crops Program (DCP), International Maize and Wheat Improvement Center (CYMMYT, Nairobi, Kenya; 7 Sustainable Agrifood Systems (SAS), International Maize and Wheat Improvement Center (CIMMYT), Nairobi, Kenya; 8 Crop Improvement, International Potato Center (CIP), Nairobi, Kenya

## Abstract

**Key message:**

Our scalable two-step method estimates genotypic performance and genetic parameters from ranking data, producing reliable results comparable to quantitative analyses, enabling the integration of ranking data into breeding pipelines.

**Abstract:**

Plant breeding research has chiefly relied on on-station experiments to evaluate varietal performance. Nevertheless, these trials often fail to represent on-farm growing conditions and farmers’ preferences, potentially leading to poorly defined breeding targets. Recent work has demonstrated the potential of using on-farm verification trials combined with ranking data to support farmers in evaluating varieties while providing information that is representative of farmers’ needs. Despite this potential, scalable methods for quantifying genetic differences and assessing the strength of the genetic signal in such trials remain limited. Here, we present a two-step procedure for analyzing trials based on ranking data, allowing the estimation of genetic parameters. The approach follows a common strategy in quantitative genetics, in which parameters are estimated from tables of genotypic means and their variances. In our framework, these estimates are obtained from Thurstonian and/or Plackett-Luce models, which treat rankings as observations of an underlying continuous trait associated with genotypic performance. Using simulated data, we showed that genotypic mean estimates derived from ranking analyses are linearly related to those obtained from quantitative trait analyses and that their variances adequately capture estimation uncertainty. We further demonstrated that incorporating these estimates and their variances into a second-step mixed-effects model yields accurate estimates of variance components. Analyses of groundnut, maize, and sweetpotato datasets confirmed the applicability of the approach and showed that ranking data can provide reliable estimates of genetic parameters. We argue that this framework can be scaled to obtain genotypic performance estimates from multi-trial on-farm data.

**Supplementary Information:**

The online version contains supplementary material available at https://doi.org/10.1007/s00122-026-05319-1.

## Introduction

Plant breeders need to assess newly developed genotypes, which is typically done using on-station trials, involving intensive phenotyping protocols and covering a limited set of environments. On-farm testing serves as a complementary approach, allowing genotype performance and farmer preferences to be assessed under actual growing conditions (Werner et al. 2025; Worku et al. 2020). On-farm testing is widely used, but is especially critical for breeding programs that target diverse, and low-input environments. Recently, a scalable citizen-science-based approach has been developed as a cost-effective method for on-farm testing of genotypes (van Etten et al. 2019a). This approach, known as the triadic comparisons of technologies (tricot), treats each farm as an incomplete block, where the performance of three genotypes is ranked by the farmer from the best to the worst. A group of sampled farms forms a tricot trial in which genotypes are allocated according to an A-optimal design, ensuring a well-connected network of pairwise comparisons across farms and maximizing the precision of genotype effect estimates (De Sousa et al. 2024; Nishii 1993). The tricot approach is easy to implement for medium- and small-scale farmers and improves reliability in evaluating traits such as yield, color, taste, and marketability. While tricot has proven effective in helping farmers and consumers evaluate and report genotypic performance, the information generated by these trials has not been fully exploited by plant breeders. This is partly due to the lack of scalable and accessible analytical frameworks for conducting quantitative genetic analyses of ranking data, as well as the limited number of studies exploring the use of ranking data in plant breeding. Consequently, a ranking-based approach remains underutilized for assessing genetic progress, evaluating trial reliability, and supporting decision-making regarding variety release.

Ranking data cannot be directly analyzed using conventional quantitative genetic methods because they do not constitute continuous quantitative phenotypic observations. Although generalized linear models can be applied to ranking responses, they become computationally demanding when the number of items (varieties in our case) is large and each individual (farmer in our case) evaluates only a subset of those items (Turner et al. 2020). An alternative is provided by order-statistics models, which treat observed rankings as realizations of an underlying continuous trait, allowing the estimation of genotypic performance. Two commonly used models, Thurstone Case V (‘Thurstonian model’ hereafter) and Plackett-Luce, adopt this approach by assuming, respectively a normal and a Gumbel distribution (type I extreme value), for the underlying trait (Luce 1959; Thurstone 1927; Yu et al. 2019). The Thurstonian and Plackett-Luce models belong to the broader class of random utility and discrete choice models, which originated in psychometrics and choice theory (Henderson 2022; Yellott 1977). Recent applications include integrating phenotypic data collected on different measurement scales (Simko and Pechenick 2010; Simko and Piepho 2011). In particular, the Plackett-Luce model has been employed to evaluate and summarize relative genotypic performance from farmer-generated rankings (de Sousa et al. 2021; van Etten et al. 2019b), although it has not been extended to the estimation of genetic parameters. In contrast, the Thurstonian model is a more natural choice based on classical quantitative genetics, where continuous traits are typically assumed to follow a normal distribution (Falconer 1989). This model has been incorporated into Bayesian mixed-model frameworks for the quantitative genetic analysis of ranked traits (Gianola 1982; Ricard and Legarra 2010), primarily in the context of animal breeding (Varona and Legarra 2020) with limited applications in crop experimentation (Halekoh and Kristensen 2008). Despite its potential for application in plant breeding, current implementations of the Thurstonian model remain computationally demanding and difficult to integrate into standard analytical pipelines.

For instance, researchers and practitioners in plant breeding often use tables of estimated genotypic means and their variances as the main input for quantitative genetic analyses, as this facilitates the aggregation and simultaneous analysis of multiple trials. Although this approach may lead to some loss of information (Möhring and Piepho 2009), it is well-suited to on-farm experimentation, where trials are often decentralized and involve multiple institutions, coordinators, farmers, researchers, and agroecological zones. This is precisely the case for tricot trials. However, an analysis based on a two-stage approach has not yet been implemented or validated for experiments with ranking data.

In this study, we propose and validate a simple and scalable two-step procedure for using ranking data as input for a quantitative genetic analysis of on-farm trial data. Currently, tricot trials data analysis is typically reported in tables of estimated genotypic means and their associated variances, derived from ranking data analyzed with the Plackett-Luce model. Similar tables are generated in quantitative traits’ analysis and have been successfully used in downstream analyses to estimate genetic parameters from aggregated trials (networks) using a two-stage approach (Smith et al. 2001; Verbyla 2023). Our procedure follows the same principle, except that in the initial step, the tables of estimated genotypic means and variances are obtained from ranking data analysis and expressed on an underlying continuous scale. After obtaining these estimates, the procedure for estimating genetic parameters, such as heritability, genetic standard deviation, and genetic correlations, based on linear mixed models is close to the analysis of quantitative traits.

We propose that our approach for analyzing experiments with ranking data can perform comparably to conventional quantitative-trait analysis, provided that the genotypic mean estimates derived from the ranking data and their associated variation are reliable. To test under which experimental conditions both criteria are met, we simulate and analyze tricot data for which the link between the underlying continuous trait and the ranking data is known. This analysis includes a comparison between the Thurstonian model, which meets the assumption of normality typically required for most quantitative trait analyses, and the Plackett-Luce model, which is the default for analyzing ranking data in tricot. Lastly, we evaluate our approach on observed data in which yield was measured and ranked on the same farms.

Our objectives are: (1) to define a robust and scalable two-step procedure for quantitative genetic analysis of ranking data; (2) to evaluate the performance of the estimates produced by this framework through a simulation study; and (3) to compare estimates based on ranking and quantitative data for the same trait using observed datasets. We thereby aim to lay the methodological foundation for incorporating ranking data from on-farm variety trials into pipelines for the analysis of single and multiple trials. This will give plant breeders a tool for reliably estimating on-farm genotypic performance and variability, helping guide decisions that respond to farmers’ needs.

## Material and methods

### Tricot trials

The motivation of our contribution is to enable the use of ranking data in plant breeding, specifically because of the prevalence of this type of data in the tricot approach. Tricot follows a citizen science approach for on-farm testing, where each farm is treated as an incomplete block. On each farm, three genotypes drawn from a larger pool are cultivated in separate plots and evaluated by the farmer, who ranks them from best to worst, based on observed performance. These evaluations are conducted independently for multiple traits, including overall performance. Limiting comparisons to three genotypes simplifies the evaluation process and reduces error, providing a practical way for small- and medium-scale farmers to give feedback to breeders and scientists (De Sousa et al. 2024). Tricot uses a forced-choice approach, meaning that no ties are allowed. Each block is observed and evaluated by the farmer who manages it, ensuring that the data reflects direct field experience. Repeated evaluations during the crop cycle can be conducted, but each block corresponds to one evaluator. Tricot trials follow an incomplete block design in which genotype allocation is generated using an A-optimality criterion to maximize the precision of genotype effect estimates. This typically results in approximately balanced genotype replication and pairwise genotype co-occurrence across farms (Bailey and Cameron 2009). The design is generated subject to the number of participating farms and the number of genotypes to be evaluated.

### Thurstonian model for ranking data analysis

For a quantitative genetic analysis based on ranking data, we focus on probabilistic models that enable continuous estimation of genotypic performance from observed rankings. Within this class of models, random utility models provide a suitable framework (Manski 1977), with the Thurstonian model being one of the earliest formulations. Under the Thurstonian model, rankings are treated as outcomes of a latent process in which the observed ranks represent underlying continuous utilities. In a given occurrence, the ordering of these utilities is directly linked to the observed ranking (Thurstone 1927; Yu et al. 2019). These utilities follow an associated probabilistic distribution whose parameters can be estimated from observed rankings, enabling subsequent quantitative genetic analyses.

Building on this framework, we adapt the Thurstonian model for the analysis of trials in which genotypes are evaluated using ranking data. We define a trial as a group of farms with similar biophysical conditions evaluated within the same season, such that environmental conditions are relatively homogeneous. Farms are treated as incomplete blocks in which only a few genotypes are tested (e.g., three genotypes in tricot experiments), making genotype × farm interactions difficult to estimate. Increasing the number of replicates within farms is generally not feasible because individual farms cover limited planted areas. Consequently, under our definition of a trial and the characteristics of the participating farms, genotype × environment interactions, including genotype × farm interactions, are expected to be small and absorbed into the residual term, together with other unexplained sources of variation.

Ranking data do not allow estimation of non-genotypic main effects because rankings are invariant to shifts in the response scale. For example, two farmers evaluating the same genotypes will produce identical rankings even if one farm consistently achieves yields that are 0.5 $t\,\textrm{ha}^{-1}$ higher than the other. Following these considerations, the Thurstonian model can be specified by defining a continuous variable $y_{jk}$ that represents the utility of genotype *j* (j=1,2,…,v), as assessed by the evaluator/farmer *k* (k=1,2,…,t):

1 $$\begin{aligned} y_{jk}=\mu _j+\epsilon _{jk} \end{aligned}$$

where $\mu _j$ is the mean utility of genotype *j* across all evaluations, and $\epsilon _{jk}$ is a random residual component capturing perception error, plot-to-plot variation, and other sources of variation not explained by the genotype mean. Under the Thurstonian model, $\epsilon _{jk}$ is normally distributed with mean 0 and variance $\sigma _\epsilon ^2$. From this point forward, we refer to the mean utilities of genotypes $\mu _1,\mu _2,\ldots$, as genotypic utility means. The probability *P* of obtaining a particular ranking $R_k$ under the Thurstonian model is defined using an order statistic model (Yu et al. 2019). We illustrate this probability with a simple example based on a tricot experiment. Consider a set of genotypes $\text {geno}\ 1,\text {geno}\ 2,\ldots , \text {geno}\ v$. Suppose farmer *k* evaluates an incomplete block $S_k$ containing three of these genotypes: $\{\text {geno}\ 1,\text {geno}\ 5,\text {geno}\ 8\}$. Farmer *k* provides the ranking $R_k: \text {geno}\ 5>\text {geno}\ 8>\text {geno}\ 1$, meaning that geno 5 was the best and geno 1 the worst. Following Böckenholt (1992), this probability is defined as a function of the underlying latent utilities as follows:

2 $$\begin{aligned} \begin{aligned}&P(R_k:\text {geno }5> \text {geno }8> \text {geno }1 \mid \\&\quad S_k=\{\text {geno }1,\text {geno }5,\text {geno }8\}) \\&\quad = P(y_{5k}>y_{8k}>y_{1k}) \\&\quad = P\!\left\{ (y_{5k}-y_{8k}>0)\cap (y_{8k}-y_{1k}>0)\right\} \end{aligned} \end{aligned}$$

The joint distribution of $y_k=(y_5k,y_8k,y_1k)'$ follows a multivariate normal distribution with mean vector $\mu _{y_k}$ and covariance matrix $\Sigma _{y_k}$. By making the change of variables $U_{1k}= y_{5k}-y_{8k}, U_{2k}= y_{2k}-y_{1k}$, the joint distribution $U_{k}=(U_{1k},U_{2k})'$, follows a bivariate normal distribution with mean vector $\mu _{U_k}$ and covariance matrix $\Sigma _{U_k }$. This definition of $U_k$ is valid for any set of genotypes.

Thus, probability P can be calculated as:

3 $$\begin{aligned} P(R_k \mid S_k )= &  P{(U_{1k}>0)\cap (U_{2k}>0)}\nonumber \\= &  \int _{0}^{\infty }\!\!\int _{0}^{\infty } \phi \!\left( U_k;\,\mu _{U_k},\,\Sigma _{U_k}\right) \,dU_{1k}\,dU_{2k} \end{aligned}$$

where ϕ is the density function of a bivariate normal distribution. For the example described, the corresponding specifications of $\mu _{U_k}$ and $\Sigma _{U_k}$ are provided in Section 1.1 of the Supplementary Information. For the general definition of P for comparisons of more than three items refer to Böckenholt (1992) and Tavernier (1991).

#### Estimation of utility-based genotypic means, variances, and quasi-variances

The probability *P* of a ranking occurrence, as defined above, is used to construct the log-likelihood function l for estimating the genotypic utility means $\hat{\mu }_1, \hat{\mu }_2,\ldots ,$. These estimations are obtained from the maximization of ℓ.

4 $$\begin{aligned} \ell (\mu _1,\mu _2,\ldots ,\mu _v) = c+\sum _{k=1}^{t} \log \!\left( P(R_k \mid S_k)\right) \end{aligned}$$

where *c* is a constant, $R_k$ is the ranking provided by farmer *k*, and $S_k$ is the subset of genotypes evaluated by farmer *k*. To ensure identifiability, two restrictions are imposed. First, $\mu _1=0$, where genotype 1 is typically chosen as a check variety. This restriction establishes a reference point and places all genotypic utility mean estimates on a relative scale, such that they are interpreted as contrasts with the check variety (Thurstone 1927; Böckenholt 1992; Turner et al. 2020). Second, the variance of the random effect of Eq. (3) is fixed to $\sigma _\epsilon ^2=1$. Fixing this variance to 1 expresses utilities in units of residual standard deviation, Gianola and Simianer (2006). This interpretation links the underlying utility scale to the quantitative metric scale of the same trait. For example, between the analysis of yield ranked and yield measured in tons per hectare ($t\,\textrm{ha}^{-1}$), the utilities assumed by the Thurstonian model are given in units of residual standard deviation of yield in $t\,\textrm{ha}^{-1}$.

As will be discussed later, both the estimates of genotypic utility means and their associated variances are crucial for estimating genetic parameters with ranking data. In the Thurstonian model, obtaining the variance-covariance matrix of the genotypic utility mean estimates, $\hat{\Sigma }_{(\hat{\mu }_1,\hat{\mu }_2,\ldots )}$ is not straightforward, due to the complexity of partial derivatives of ℓ. To address this, we use an asymptotic approximation based on the observed Fisher information matrix (Efron and Hinkley 1978), obtained from the Hessian matrix, which is derived within the maximization of ℓ.

Variances for the genotypic utility mean estimates are obtained directly from the diagonal elements of $\Sigma _{(\hat{\mu }_2,\hat{\mu }_3,\ldots )}$. However, due to the constraint imposed on the reference genotype $\mu _1=0$, only v-1 variances are estimated, and these estimates depend on the choice of reference genotype. In other words, if we change our genotype reference, the estimates for all variances will change. Quasi-variances (*qV*) provide an alternative approach for expressing the uncertainty associated with genotypic utility mean estimates and facilitate inference for arbitrary contrasts among genotypes (Ridout 1989; Turner et al. 2020; Firth 2003). The underlying idea is that the variance of any contrast between genotypes can be approximated by the sum of their corresponding quasi-variances. Thus, for a contrast that represents any pair of genotypes *j* and *k*,

5 $$\begin{aligned} \hat{V}(\hat{\mu }_j-\hat{\mu }_k) \approx \widehat{qV}(\mu _j ) + \widehat{qV}(\mu _k ) \end{aligned}$$

where $\hat{V}(\hat{\mu }_j-\hat{\mu }_k)$ is the estimated variance of the contrast between the utility means of genotypes *i* and *j*, and $\widehat{qV}(\hat{\mu }_j )$ and $\widehat{qV}(\hat{\mu }_k )$ are the corresponding estimated quasi-variances. Unlike conventional estimated variances obtained under a reference-category parameterization, quasi-variances are largely invariant to the choice of reference genotype, provide uncertainty measures for all genotype estimates (including the reference genotype), and provide a compact approximation to the information contained in the variance-covariance matrix. Quasi-variance estimates are obtained by minimizing the objective function in Eq. 6 (see Firth 2000, for details).

6 $$\begin{aligned} \sum _{j<k} \left[ \log \!\left( \widehat{qV}(\hat{\mu }_j) + \widehat{qV}(\hat{\mu }_k) \right) - \log \!\left( \widehat{V}(\hat{\mu }_j-\hat{\mu }_k) \right) \right] ^2 \end{aligned}$$

where $\widehat{V}(\hat{\mu }_j-\hat{\mu }_k)$ is obtained from the estimated variance–covariance matrix $\widehat{\Sigma }_{(\hat{\mu }_2,\hat{\mu }_3,\ldots )}$ as $\widehat{V}(\hat{\mu }_j-\hat{\mu }_k) = \widehat{V}(\hat{\mu }_j) + \widehat{V}(\hat{\mu }_k) - 2\,\widehat{\operatorname {Cov}}(\hat{\mu }_j,\hat{\mu }_k)$. For contrasts involving the reference genotype, $\widehat{V}(\hat{\mu }_j-\hat{\mu }_1) = \widehat{V}(\hat{\mu }_j)$, since $\hat{\mu }_1$ is fixed at zero.

### Comparison with the Plackett-Luce models

The Plackett-Luce model has so far been the default approach for analyzing tricot data and follows the same principle as the Thurstonian model in defining underlying utilities associated with the ranked items (de Sousa et al. 2021; Brown et al. 2022). Similarly, these utilities can be expressed as a function of a mean utility and a random error, equivalent to Eq. (1). The advantages of the Plackett-Luce model are that it facilitates computation, that it obeys the Luce Axiom (independence of irrelevant alternatives), and that it is available in many tools for data analysis, including an R package (Turner et al. 2020).

The main difference of relevance here is that the Plackett-Luce model assumes that $y_{jk}$ follows a Gumbel distribution (type I extreme value), while the Thurstonian model assumes a normal distribution (Allison and Christakis 1994; Yellott 1977; Turner et al. 2020). This affects the scale of the estimates derived from the model. If the underlying trait is normally distributed, as is typically assumed in quantitative genetics, estimates from the Plackett-Luce model are biased. This bias could be corrected by a scaling factor but this depends on the genetic-to-residual standard deviation ratio and on the number of replicates (Supplementary Section 1.1 and Fig. S1). In contrast, for traits with an underlying normal distribution, estimates from the Thurstonian model are unbiased and do not depend on any additional parameters. Comparisons of the estimates derived from these two models for ranking data analysis are presented in the Results section, and a scaling factor to correct Plackett-Luce estimates when the underlying trait is normally distributed is discussed in Supplementary Section 1.3.

### Our framework for quantitative genetic analysis of ranking data

**Fig. 1:**
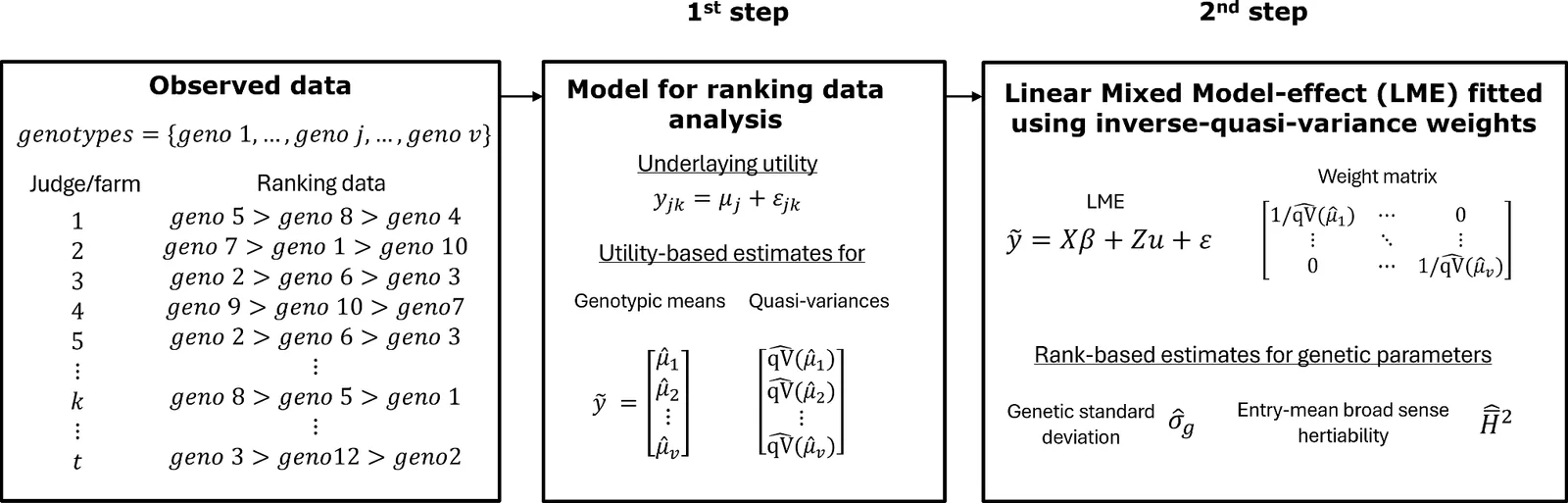
Two-step procedure for estimating genotypic means, genetic standard deviation, and heritability using ranking data. Here, $\tilde{y}$ is a vector of genotypic utility mean estimates; β and *u* are vectors of fixed and random effects, with design matrices, *X* and *Z* respectively; and ϵ is the residual vector

We adapt a two-stage approach commonly used in quantitative genetic analyses (Piepho et al. 2012a; Verbyla 2023) to a framework that handles ranking data. In the first step, genotypic means and quasi-variances (*qV*) are estimated on the utility scale. These estimates are then used in a linear mixed-effects model to estimate genetic parameters in the second step (Fig. 1). We illustrate our framework using a single-environment trial, but it can be readily extended to multi-environment analyses, provided that tables of genotypic means and their corresponding *qV* estimates are available for each environment.

In the first step of our framework, we move from a discrete ordinal space to a continuous one, using the ranking data as an input that allows to quantify the performance of the genotypes evaluated. Here, we use the Thurstonian model to derive the genotypic utility mean estimates $\hat{\mu }_1, \hat{\mu }_2, \ldots , \hat{\mu }_v$ along with their corresponding quasi-variances $\widehat{qV}(\hat{\mu }_1), \widehat{qV}(\hat{\mu }_2), \ldots$ In the second step, these estimates are used to fit the following linear mixed-effect model.

7 $$\begin{aligned} \tilde{y} = X\beta + Zu + \delta \end{aligned}$$

where $\tilde{y}$ is a vector of estimated genotypic utility means, β and *u* are vectors of fixed and random effects, with design matrices, *X* and *Z*, respectively, and δ is the residual vector. The random effects *u* and δ follow multivariate normal distributions with mean zero and covariance matrices $\sigma _g^2I$ and $\sigma _\delta ^2I$, respectively. This model can include multiple genotype-level fixed effects such as release year, entry type category, or origin of the entry, but when none are specified, it contains only a fixed intercept. The vector *u* contains the random effects of the genotypes. The mixed model equations are:

8 $$\begin{aligned} \begin{bmatrix} X^{\top }V^{-1}X & X^{\top }V^{-1}Z \\ Z^{\top }V^{-1}X & Z^{\top }V^{-1}Z + \sigma _g^{-2}I \end{bmatrix} \begin{bmatrix} \beta \\ \hat{u} \end{bmatrix} = \begin{bmatrix} X^{\top }V^{-1}\hat{\mu } \\ Z^{\top }V^{-1}\hat{\mu } \end{bmatrix} \end{aligned}$$

where $V^{-1}$ is a diagonal weight matrix whose elements are the inverses of the quasi-variance estimates (*qV*). Quasi-variances are used instead of conventional variance estimates because they summarize the information contained in the full variance-covariance matrix and quantify the uncertainty associated with all genotype estimates, including the reference genotype, as described in  Estimation of utility-based genotypic means, variances, and quasi-variances section. Consequently, defining $V^{-1}$ based on *qV* estimates provides a coherent framework for obtaining valid estimates of the genetic parameters derived from the model in Eq. (7). Comparisons of genetic standard deviation estimates obtained using *qV* versus conventional variance estimates as weights are presented in Fig. S2. An alternative definition of the weight matrix, based on the full variance-covariance matrix, is mentioned in the Discussion.

From the second step onward, the conventional analysis for quantitative traits can be implemented using Eq. (7) and Eq. (8), enabling the estimation of Best Linear Unbiased Predictions (BLUPs) and variance components. We did not include genotype × environment and/or genotype × year effects in our method, as addressing these factors would have considerably expanded the scope and complexity of this study. However, our approach can be extended to a multi-trial analysis by aggregating tables of genotypic utility mean estimates and their variances in a manner similar to that of Piepho et al. (2012a) and Verbyla (2023), thereby enabling the incorporation of environment and year effects.

#### Estimation of genetic standard deviation, heritability, and genetic correlations using ranking data

The genetic standard deviation $\sigma _g$ is estimated using restricted maximum likelihood (REML). A detailed description of its estimation within the two-stage approach framework is provided by Verbyla (2023).

Heritability represents the proportion of phenotypic variation attributable to genetic differences among individuals within a given population and environment, and is commonly used to assess trial precision and the potential response to selection (Falconer 1989). Narrow-sense heritability measures the proportion of phenotypic variance explained by additive genetic variance, whereas broad-sense heritability measures the proportion explained by total genetic variance, including additive and non-additive effects (Barry et al. 2023). In this study, we focus on broad-sense heritability because tricot is primarily applied in late-stage trials, where the objective is to evaluate the consistency of differences among advanced breeding lines and identify superior genotypes, rather than predict the breeding value of potential parents. Moreover, estimating narrow-sense heritability typically requires pedigree or genomic information to separate additive from non-additive genetic effects, data that are often unavailable in tricot datasets.

Broad-sense heritability was estimated following Cullis et al. (2006), which is particularly suitable for quantitative genetic analyses based on ranking data. As noted by Frick (2023), utility mean estimates derived from ranking models are associated with heterogeneous levels of uncertainty. The Cullis heritability can account for this feature by using the average variance of pairwise differences between the best linear unbiased predictors (BLUPs) of genotypic effects, which provides a robust measure of trial precision in the presence of complex variance structures.

### Simulation study

We evaluate the performance of our framework in estimating genotypic means, their standard errors, genetic standard deviations, and heritability using simulated tricot data. For each plot, observed yield (in $t\,\textrm{ha}^{-1}$) and corresponding rank-based evaluations are generated. The simulation, estimation, and analysis procedures are described below.

1. We generate observations of maize grain yield in $t\,\textrm{ha}^{-1}$ following the function,
  9 $$\begin{aligned} \text {Yield}_{jk} = \text {grand mean} + \text {genotype}_j + \text {farm}_k + \text {residual}_{jk} \end{aligned}$$
  here $\text {Yield}_{jk}$ is the simulated yield of the *j*th genotype (j=1,2,…,v) on the *k*th farm (k=1,2,…,m) and the *jk*th plot; grand mean is the grand mean of yield; and $\text {genotype}_j$, $\text {farm}_k$, and $\text {residual}_{jk}$ are random deviations for the *j*th genotype, *k*th farm, and *jk*th plot. These deviations are assumed to be normally distributed with mean zero and standard deviations $\sigma _{\text {geno}}$, $\sigma _{\text {farm}}$, and $\sigma _{\text {res}}$, respectively. We define $\text {mean}_j=\text {grand mean} +\text {genotype}_j$ as the true genotypic mean for the *j*th genotype, and $\sigma _{\text {geno}}$ as the true genetic standard deviation of the trial.
2. Within each farm, the three simulated yield values for each plot are ranked from 1 to 3, where 1 is the highest yield, and 3 is the lowest yield. Notice that once the simulated observed yields are converted to ranks, the main farm effect is inherently removed from the ranking analysis.
3. We split the data analysis for quantitative metric (yield in $t\,\textrm{ha}^{-1}$) and ranking data to estimate genotypic means and their corresponding covariance matrix (first step).
  For quantitative metric data on yield, we estimate Best Linear Unbiased Estimates (BLUEs) using the conventional Linear Mixed Model (LMM) approach, treating genotype as a fixed effect, and farm and residual variation as random effects.
  For ranking yield data, we estimate the genotypic utility means using the Thurstonian and Plackett-Luce models.

For both analyses, the estimated covariance matrix of the estimated genotypic means was obtained from the observed Fisher information matrix.

In the second step, for both analyses, the genetic standard deviation and heritability are estimated using an LMM, where genotypic means are modeled as a function of a fixed intercept and random effects for genotype and residual variation (see Eq. (7)).

To ensure consistency between analyses based on quantitative and ranking data, the weight matrix used in the second step is always diagonal, with entries equal to the inverses of the quasi-Variances (*qV*) of the genotypic utility mean estimates. These *qV* are computed from the observed Fisher information matrix using the R function qvcalc().

To distinguish the estimates obtained from each analysis, we use the term ‘quantitative-based estimates’ for the analysis of the quantitative metric of yield ($t\,\textrm{ha}^{-1}$) and ‘utility-based estimates’ for those derived from the analysis of ranking data. Finally, the quantitative- and utility-based estimates are compared with their reference values. These reference values include the true genotypic means and genetic standard deviation, taken directly from the parameters used in the simulation, while for heritability, these values are obtained from a one-step LMM analysis of yield in $t\,\textrm{ha}^{-1}$ (Cullis et al. 2006; Smith et al. 2001).

To ensure comparability with utility-based estimates, all parameters and estimates in $t\,\textrm{ha}^{-1}$ were converted to units of true residual standard deviation ($\sigma _{\text {res}}$). Each genotypic mean in $t\,\textrm{ha}^{-1}$, either true or estimated, was centered by subtracting the reference genotypic mean and scaled by dividing by $\sigma _{\text {res}}$. Similarly, the genetic standard deviation is divided by $\sigma _{\text {res}}$.

We assess the accuracy of our utility-based estimates by comparing them to their reference values using performance metrics and scatterplots. For the estimated genotypic means, we use the Kendall correlation coefficient, which is robust to outliers and sensitive to ordering. Further, we look at bias and Root Mean Square Error (RMSE). For the estimated genetic standard deviation and heritability, the assessment is conducted using scatterplots.

#### Simulation of multiple tricot trials using different values of variance components and numbers of replicates

To test the robustness of our framework, we simulated multiple scenarios of tricot trials with different variance component values and four sample sizes (see Table 1). The values for grand mean and $\sigma _{\text {farm}}$ were based on previous publications on on-farm yield trials for maize in Africa (Cairns et al. 2013; Das et al. 2019; Worku et al. 2020; Alam et al. 2022). The values for $\sigma _{\text {geno}}$ and $\sigma _{\text {res}}$ were also derived from the same publications, but their ranges were extended to include both lower and higher extreme values, allowing us to explore the conditions under which reliable estimates can be obtained. The average values for $\sigma _{\text {geno}}$ and $\sigma _{\text {res}}$ based on these publications are highlighted in bold. The number of genotypes tested was fixed at 20. We simulated 18480 trials, corresponding to all possible combinations of the parameters in Table 1 ($11 \times 14 \times 4$ parameter combinations, each replicated 30 times). Each trial was simulated and analyzed individually.

**Table 1:** Parameters used in the simulation of tricot trials for maize grain yield $t\,\textrm{ha}^{-1}$

Parameter	Value(s)
Grand mean (μ)	3.63
Number of genotypes (*v*)	20
Farm standard deviation ($\sigma _{\textrm{farm}}$)	3.352
Genetic standard deviation ($\sigma _{\textrm{geno}}$)	0.05, 0.1, 0.2, 0.4, 0.6, **0**.**8**, 1, 1.2, 1.5, 1.8, 2
Residual standard deviation ($\sigma _{\textrm{res}}$)	0.4, 0.6, 0.8, **0**.**9**, 1.1, 1.4, 1.6, 2, 2.5, 3, 3.5, 4, 4.5, 5
Number of replicates per genotype ($n_r$)	3, 10, 30, 100
Number of simulation replicates ($R_{\textrm{exp}}$)	30

μ denotes the grand mean of grain yield; *v* is the number of genotypes tested; $\sigma _{\textrm{geno}}$ is the genetic standard deviation; $\sigma _{\textrm{farm}}$ is the farm (location) standard deviation; and $\sigma _{\textrm{res}}$ is the residual standard deviation. $n_r$ indicates the number of replicates per genotype, and $R_{\textrm{exp}}$ denotes the number of simulation replicates. The literature-based average values for $\sigma _{\textrm{geno}}$ and $\sigma _{\textrm{res}}$, derived from published on-farm trials, are shown in **bold**

#### Validation of variances of genotypic utility mean estimates

We validated the variance obtained from the observed Fisher information matrix Estimation of utility-based genotypic means, variances, and quasi-variances, for a simulated trial with parameters ($\sigma _{\text {geno}}=0.8,\sigma _{\text {res}}=0.9,n_r=30$). These parameters are averages of literature-based values, with 30 replicates per genotype, which is common in tricot trials. Using these parameters, we simulated 100 trials with fixed true genotypic means, converting yield evaluations to ranks, and obtaining genotypic utility mean estimates and their variances for each trial. This validation was conducted using the square root of the variances (standard errors, SE). Across simulations, we calculated the standard deviation of the 20 estimated genotypic means and the mean of their SEs, then compared these results in a scatterplot.

### Application to observed data

Our observed datasets include tricot trials for groundnut, maize, and sweetpotato crops across African regions (Table 2) with both measurements, ranking, and quantitative data of yield. In all four datasets, farmers collected the ranking data, while the quantitative data were collected by technicians or trained lead farmers in the sweetpotato and groundnut trials, and by the farmers themselves in both maize trials. The datasets for groundnut and sweetpotato are available on Zenodo (de Sousa et al. 2025).

**Table 2:** Description of observed datasets of yield with quantitative and ranking data

Crop	Country	Genotypes	Replicates per genotype (mean ± 95% CI)
Groundnut	Tanzania	54	28.5±3.46
Maize (Early-maturity)	Kenya	12	66.8±2.70
Maize (Intermediate-maturity)	Kenya	25	58.7±3.95
Sweetpotato	Mozambique	8	16.5±1.69

In this analysis, the comparison between quantitative- and utility-based estimates followed the same approach used for the simulated data. In addition, the genetic correlation between the two evaluation methods was assessed by extending the model in Eq. (7) to a multi-trait model (Supplementary Section 1.4), and heritability was estimated from rankings derived directly from the observed yield ($t\,\textrm{ha}^{-1}$).

### Computational resources and software

We conducted the simulation study and the analysis of observed datasets on a Linux operating system (Ubuntu 20.04.4 LTS) with 251 GiB of memory and 96 cores. Our framework was implemented in the R software (R Core Team, 2023). In the simulation study, we used the randomize() function from the *ClimMobTools* package (Quirós et al. 2024) to generate an incomplete block A-optimal design for each trial. For data analysis, we employed the *tidyverse* package (Wickham et al. 2019), specifically using *tidyr* for data processing and *ggplot2* for data visualization. We used the *PlackettLuce* (Turner et al. 2020) and *gosset* (de Sousa et al. 2023) packages to read and process ranking data and used custom-made implementations of Thurstonian and Plackett-Luce models, available in (Dorado-Betancourt and Van Heerwaarden 2025). The optimization of likelihood functions was carried out using the Broyden-Fletcher-Goldfarb-Shanno (BFGS) algorithm implemented in the R function optim() (Head and Zerner 1985). BFGS provides an approximation of the Hessian matrix that is required to estimate variances and quasi-variances for the genotypic mean utility estimates. For the implementation of the mixed-effects models, we used the package *ASReml* (The VSNi Team, 2023).

## Results

The results are presented in three parts: (1) an analysis of estimated genotypic means and standard errors from a simulated tricot trial to illustrate the first-step of the ranking analysis; (2) an assessment of utility-based estimates across multiple simulated tricot trials, with varying genetic and residual standard deviations and numbers of replicates, validating the estimates obtained in both steps of our approach under different conditions; and (3) an application of the proposed method to observed data.

### Analysis of estimated genetic values and their standard errors with ranking data

For this analysis, we simulated a trial using the following settings ($\sigma _\text {geno}=0.8, \sigma _\text {res}=0.9,n_r=30$). These values represent the averages of literature-based estimates and the typical number of replicates used in tricot trials (see Table 1).

**Fig. 2:**
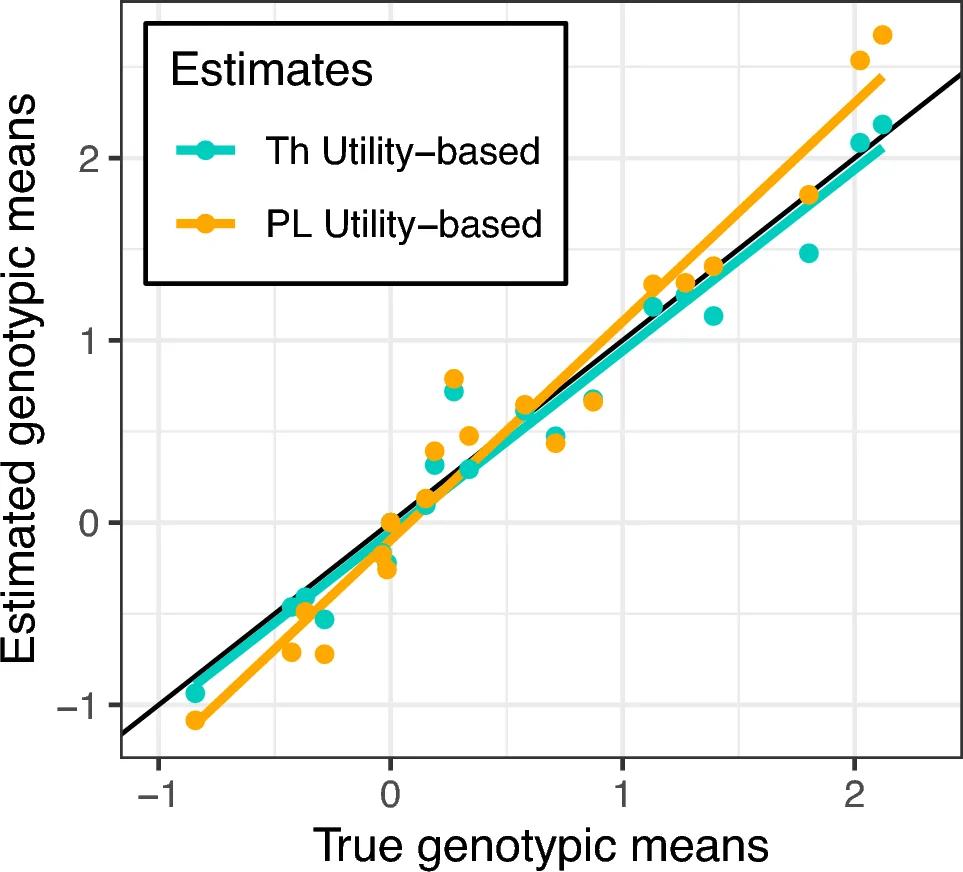
True versus estimated genotypic means for simulated grain yield in maize. The parameters used in this simulation are $\sigma _{\textrm{geno}} = 0.8$, $\sigma _{\textrm{res}} = 0.9$, and $n_r = 30$. Both axes are expressed in standardized units of $\sigma _{\textrm{res}}$. Th and PL refer to estimates obtained using the Thurstonian and Plackett-Luce models, respectively. The solid black line represents the 1:1 reference line

Figure 2 shows that the relationship between true and estimated genotypic means is linear across both models used to analyze ranking data. As expected, estimates from the Thurstonian model are unbiased because the model assumes that rankings arise from underlying utilities that follow a normal distribution, matching the simulation setup. In contrast, the Plackett-Luce model exhibits a consistent bias because it assumes that the latent utilities underlying the rankings follow a Gumbel distribution. When the true utilities are normally distributed, the variance implied by the Gumbel distribution is inflated, causing the estimated genotypic means to be biased in proportion to their absolute values. However, this bias follows a systematic pattern and can be approximately corrected by applying an appropriate scaling factor. Details are provided in Supplementary Sections 1.2 and  1.3.

Although we explored the identification of a unique scaling factor to align the estimates of both models for any case, we found that this factor is not constant, as it is affected by genetic standard deviation, residual standard deviation, and the number of replicates (see Supplementary Section 1.3 and Fig. S1). From our simulations, we deduced that when the ratio of genetic to residual standard deviation is 0.71 or lower (as might be expected in on-farm trials, and the number of replicates exceeds 30), the scaling factor typically ranges between 0.85 and 0.90.

Since we are interested in analyzing the performance of the Plackett-Luce model estimates independently of the effect of this scaling factor, all Plackett-Luce estimates were re-scaled to align with the Thurstonian scale (PL utility-based (scl)). The scaling factor was defined separately for each trial using a simple linear regression of Thurstonian estimates on PL estimates, without an intercept.

**Fig. 3:**
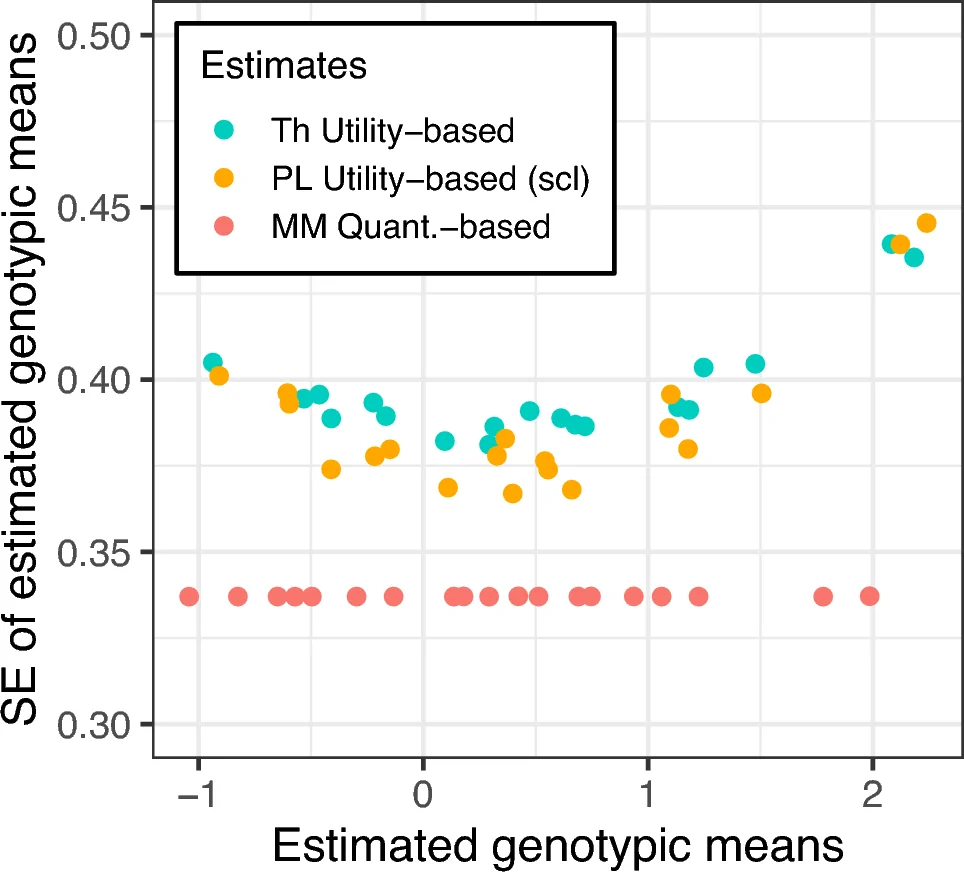
Estimated genotypic means versus their standard errors (SE) for simulated grain yield in maize. The trial parameters used in this simulation are $\sigma _{\textrm{geno}} = 0.8$, $\sigma _{\textrm{res}} = 0.9$, and $n_r = 30$. Both axes are expressed in standardized units of $\sigma _{\textrm{res}}$. Th, PL, and MM refer to estimates obtained using the Thurstonian, Plackett-Luce, and linear mixed-effects models, respectively. Utility-based estimates from the Plackett-Luce model were scaled to the same units as the Thurstonian estimates

The estimated standard errors of genotypic means from ranking data reveal three key properties (Fig. 3). (1) Contrary to estimates from quantitative data, utility-based estimates of standard errors are not constant, but follow a quadratic function of the estimated genotypic means. This occurs because when genotypes consistently outperform or underperform others in the sample, less information about their relative values in the latent space can be recovered. (2) Standard errors of utility-based estimates are considerably larger than quantitative-based estimates, reflecting the loss of information due to ranking. In fact, we show in Fig. S3 that, for most cases in the expected on-farm trial conditions, about 30% more replicates are needed in ranking trials to achieve the same precision as estimates obtained from quantitative data analysis. (3) The scaled standard errors of the Plackett-Luce model are relatively close to, yet slightly more dispersed than those from the Thurstonian model. These discrepancies may be partly attributable to the scaling factor applied to align the estimates of both models. However, differences in the intrinsic stability of the parameter estimates between models may also contribute.

**Fig. 4:**
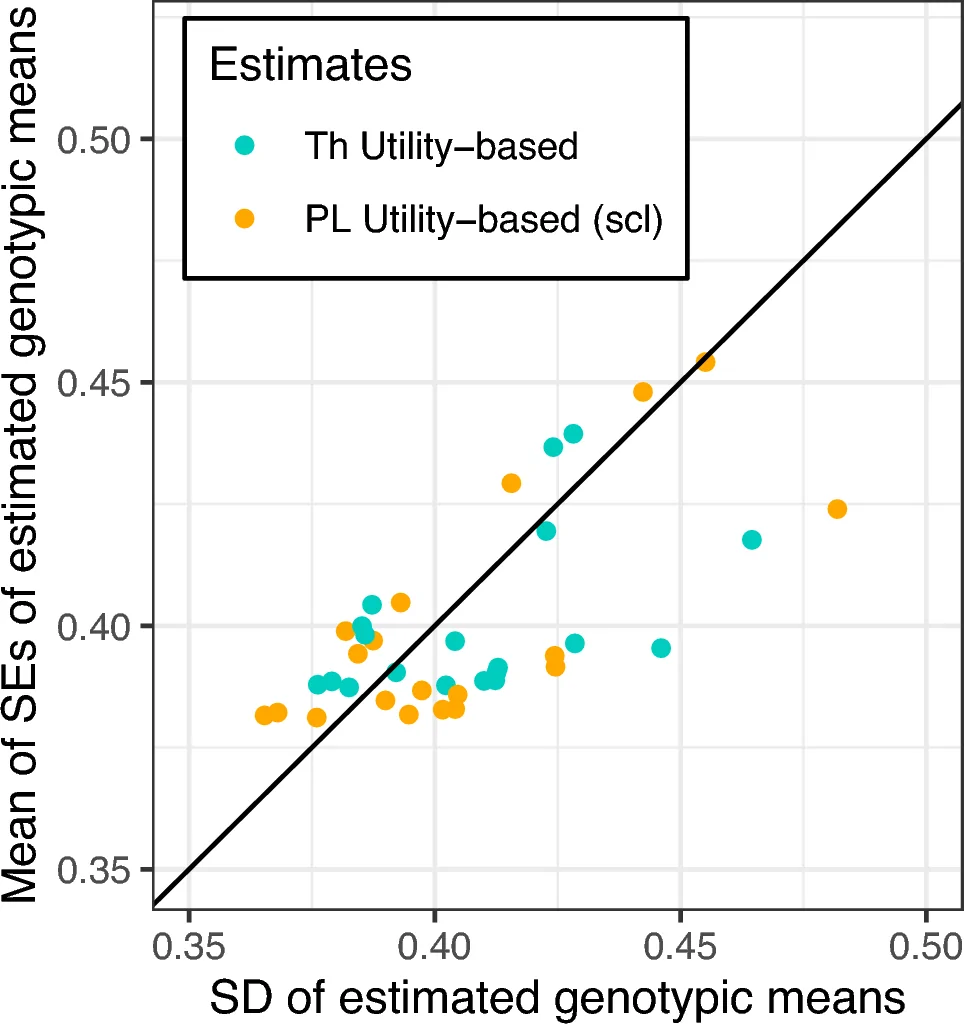
Expected variability of genotypic means versus observed standard errors for simulated grain yield in maize. Expected variability corresponds to the standard deviation (SD) of the estimated genotypic means, whereas observed variability corresponds to the mean of their standard errors (SEs), estimated using the observed Fisher information matrix. The means and standard deviations were calculated over 100 simulation replicates of a trial with $\sigma _{\textrm{geno}} = 0.8$, $\sigma _{\textrm{res}} = 0.9$, and $n_r = 30$, while keeping the true genotypic means fixed. Th and PL refer to estimates obtained using the Thurstonian and Plackett-Luce models, respectively. Utility-based estimates from the Plackett-Luce model were scaled to the same units as the Thurstonian estimates. The solid black line represents the 1:1 reference line

The validation of standard errors calculated using the observed Fisher information matrix is shown in Fig. 4. In this graph, two trials were discarded because they contained undefined estimates with extremely high or low values that affected the calculation of the expected standard error. Cases with undefined estimates are discussed in the following subsection. After removing these two cases, the validation results suggest that the variations inherent in the genotypic utility mean estimates were reasonably well captured. Nevertheless, for some genotypes, particularly those with the highest standard deviations on the right side of Fig. 4, the estimation of the standard errors was less precise, and probably more replicates are needed.

### Performance of genotypic means, genetic standard deviation, and heritability estimates

#### Genotypic means

**Fig. 5:**
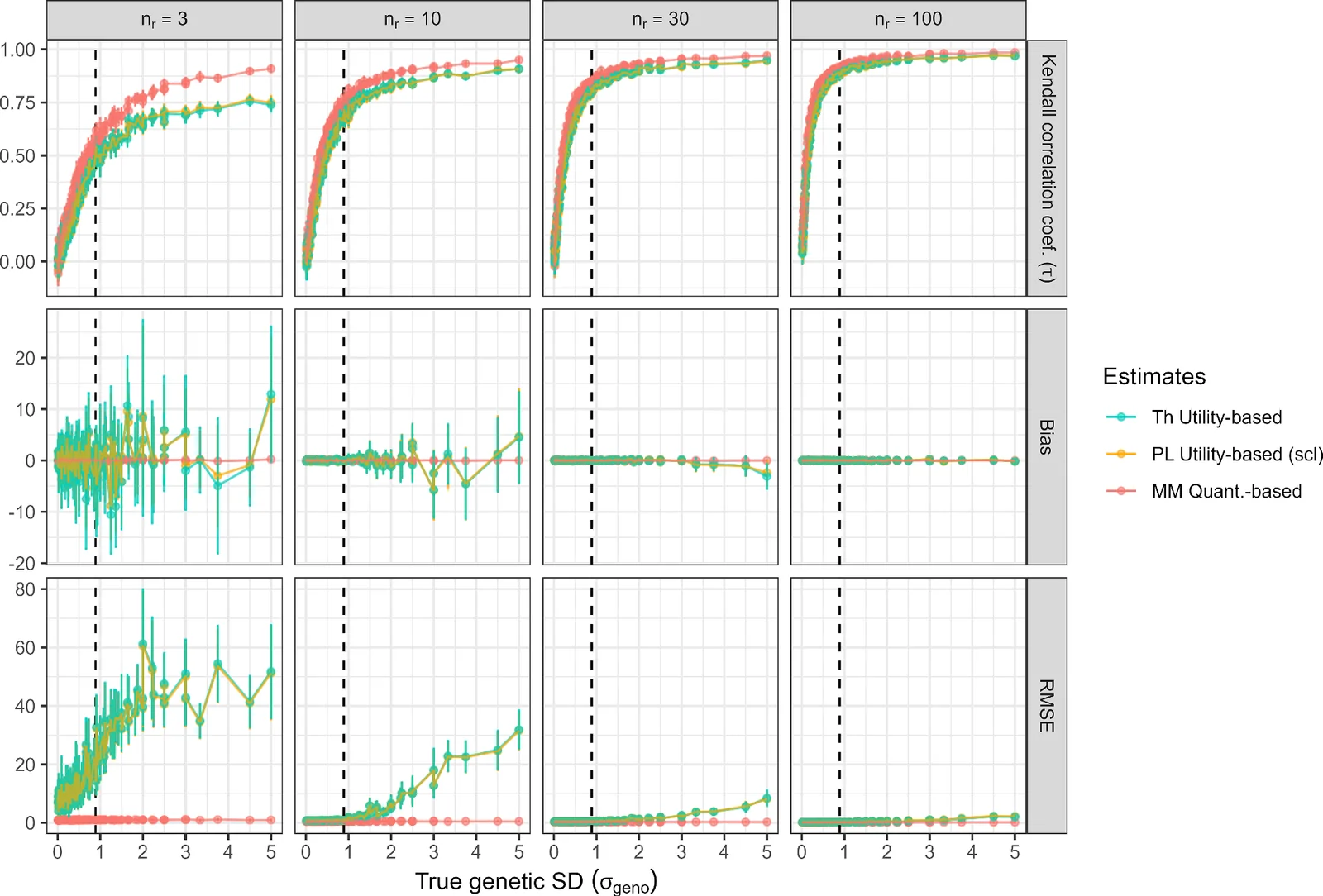
Effects of genetic variability and replication on estimated genotypic performance for simulated grain yield in maize. The top panels correspond to the four values of the number of replicates ($n_r$), whereas the right panels represent the evaluation metrics, namely, the Kendall correlation coefficient, bias, and Root Mean Square Error (RMSE), calculated from the true and estimated genotypic means, in that order. The y-axis values represent the means of the evaluation metrics calculated over 30 simulation replicates with the same parameter settings, and the corresponding error bars indicate their standard errors. Th, PL, and MM refer to values obtained using the Thurstonian, Plackett-Luce, and linear mixed-effects models, respectively. True genetic standard deviation (SD), bias, and RMSE are expressed in units of $\sigma _{\textrm{res}}$. Utility-based estimates from the Plackett-Luce model were scaled to the same units as the Thurstonian estimates. Vertical dashed lines highlight the results for a trial with $\sigma _{\textrm{geno}} = 0.8$ and $\sigma _{\textrm{res}} = 0.9$

The assessment of the estimated genotypic means in Fig. 5 reveals four key insights: (1) After correcting the Plackett-Luce estimates by the scaling factor to align them to the Thurstonian estimates (see  Analysis of estimated genetic values and their standard errors with ranking data section), the estimates from all three models are unbiased; (2) Utility-based estimates underperform compared to the quantitative-based estimates in terms of precision and correlation. However, this difference in performance is reduced when the number of replicates ($n_r$) is 30 or more; (3) An increase in genetic standard deviation with low ($n_r$) leads to higher correlation between true and estimated genotypic means, but decreases the precision of utility-based estimates; and (4) The estimates of the Thurstonian model and the Plackett-Luce (scl) model yield almost the same performance.

**Fig. 6:**
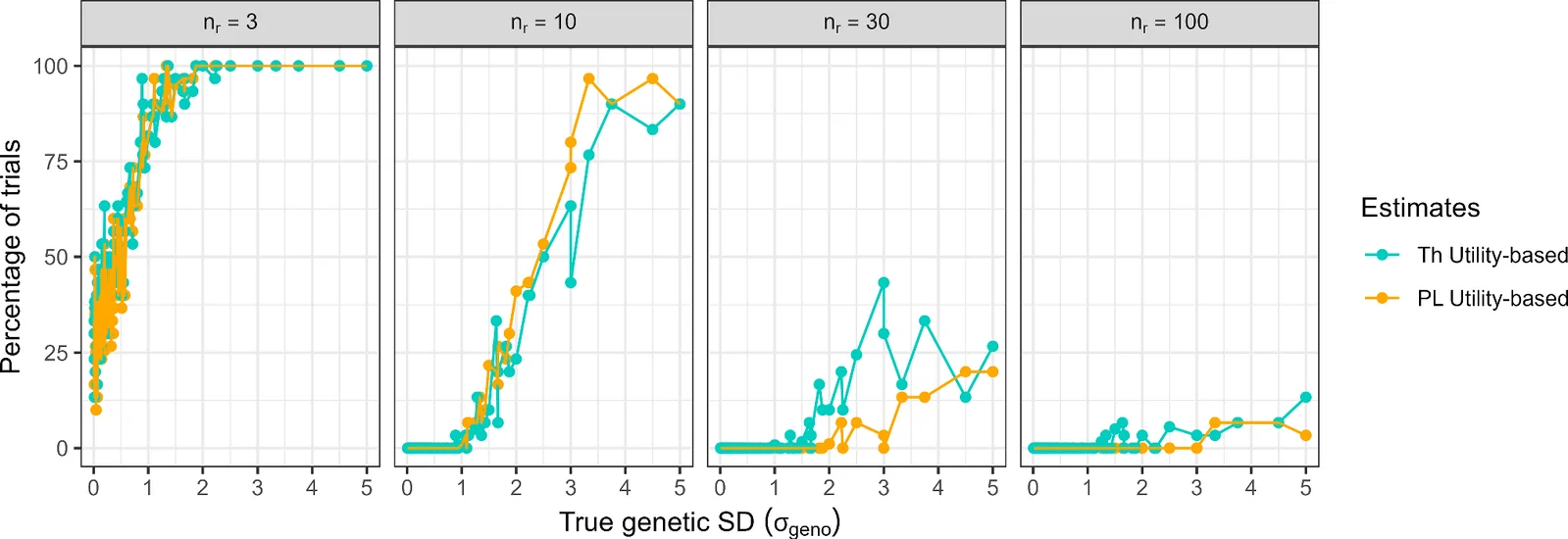
Effects of genetic variability and replication on trials with an undefined covariance matrix. Th and PL refer to estimates obtained using the Thurstonian and Plackett-Luce models, respectively. True genetic standard deviation (SD) is expressed in units of $\sigma _{\textrm{res}}$

From our simulations, we found that 17–19% of the trials produced undefined estimates for the covariance matrix of the genotypic utility mean estimates from the Thurstonian or Plackett-Luce model analyses. These cases are analyzed in Fig. 6, which shows that they mainly correspond to trials with high genetic standard deviation ($\sigma _\text {geno}$ larger than 1.5) and low number of replicates ($n_r$ lower than 30). Under these conditions, it is more likely that certain genotypes are repeatedly ranked as either consistently best or worst at farms. This invariance in ranking results in a lack of information needed to estimate continuous relative differences among genotypes. In the most extreme case, a genotype that is always ranked either first or last provides no variability in its relative continuous distance in the latent space, making it impossible to accurately estimate its genotypic value. As a result, the estimate may become extremely large (positive or negative), and the associated variance may also be very large or infinite. A detailed example is provided in Fig. S4.

#### Genetic standard deviation and heritability

**Fig. 7:**
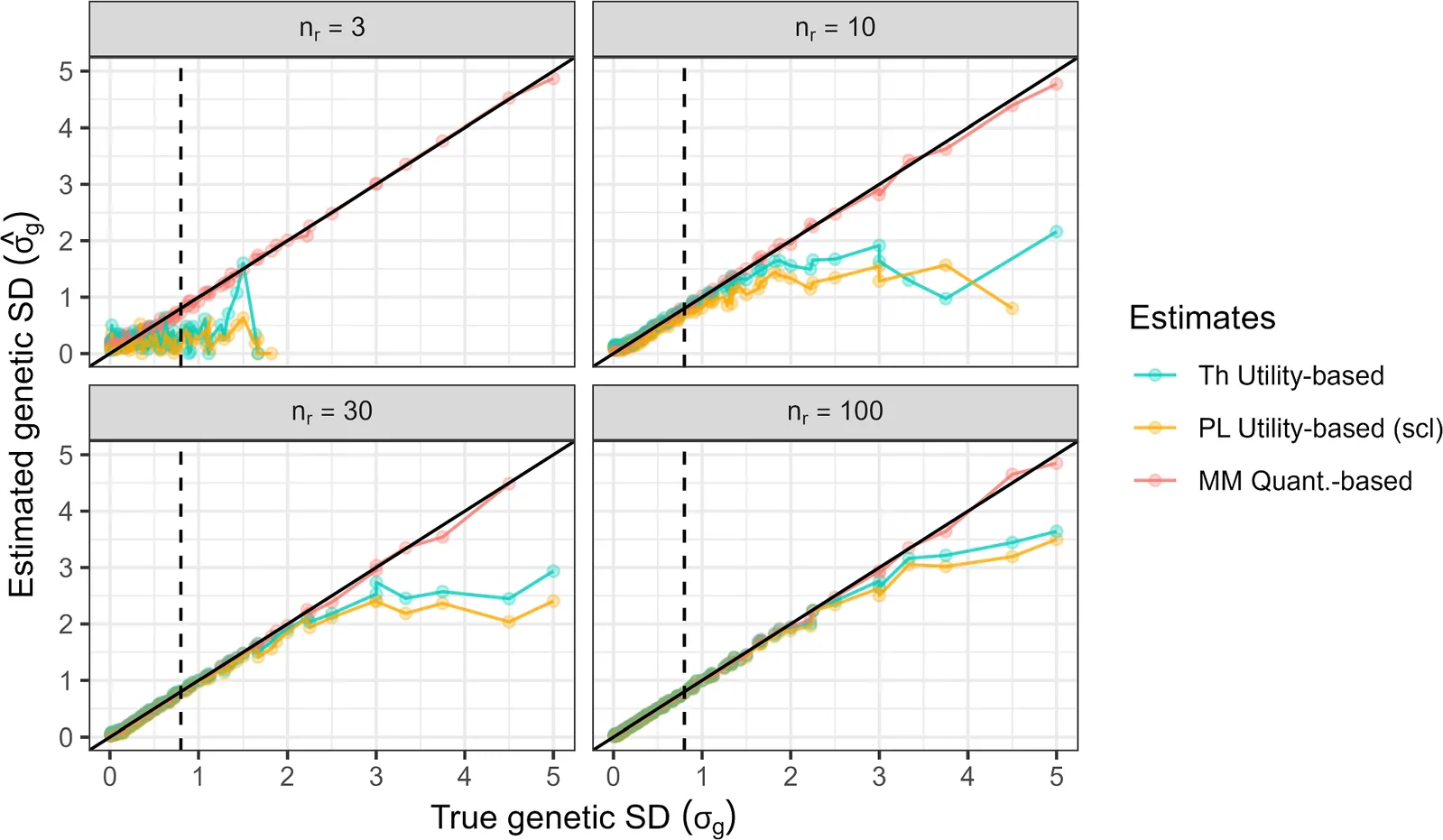
True versus estimated genetic standard deviation (SD) for simulated grain yield in maize. The top panels correspond to the four values of the number of replicates ($n_r$). The y-axis values represent the means of the estimates calculated over 30 simulation replicates with the same parameter settings (the disaggregated version of this figure is shown in Fig. S5). Both axes are expressed in standardized units of $\sigma _{\textrm{res}}$. Th, PL, and MM refer to estimates obtained using the Thurstonian, Plackett-Luce, and linear mixed-effects models, respectively. Utility-based estimates from the Plackett-Luce model were scaled to the same units as the Thurstonian estimates. The solid black line represents the 1:1 reference line. Vertical dashed lines highlight the results for a trial with $\sigma _{\textrm{geno}} = 0.8$ and $\sigma _{\textrm{res}} = 0.9$

Building on the results observed in Figs. 5 and 7 shows that accurate estimates of genetic standard deviation can be obtained when genotypic means are accurately estimated. The performance of these estimates is negatively affected as the true standard deviation increases, often leading to underestimation. In some cases, it is not possible to estimate values when the number of replicates is too low. As mentioned, these conditions produce undefined genotypic utility means estimates, resulting in undefined or inflated estimates for their respective variances. High variance estimates lead to high quasi-variances, which serve as weights in the mixed model for estimating genetic standard deviation. Consequently, genotypes with undefined estimates contribute little or nothing to the estimation of genetic standard deviation, explaining the observed underestimation. Although increasing the number of replicates can mitigate this loss of performance, more than 100 replicates may be required to obtain reliable estimates when the true genetic standard deviation exceeds 2.

The calculation of Cullis heritability yielded negative values in 17-19% of all simulated trials. These negative estimates were present across all mixed-effects models used for quantitative data analysis, as well as in Thurstonian and Plackett-Luce analyses, which suggest that this is a limitation of this method of estimating heritability. Such cases were particularly associated with extremely low genetic standard deviations combined with high residual standard deviations. These cases were excluded from the analysis.

**Fig. 8:**
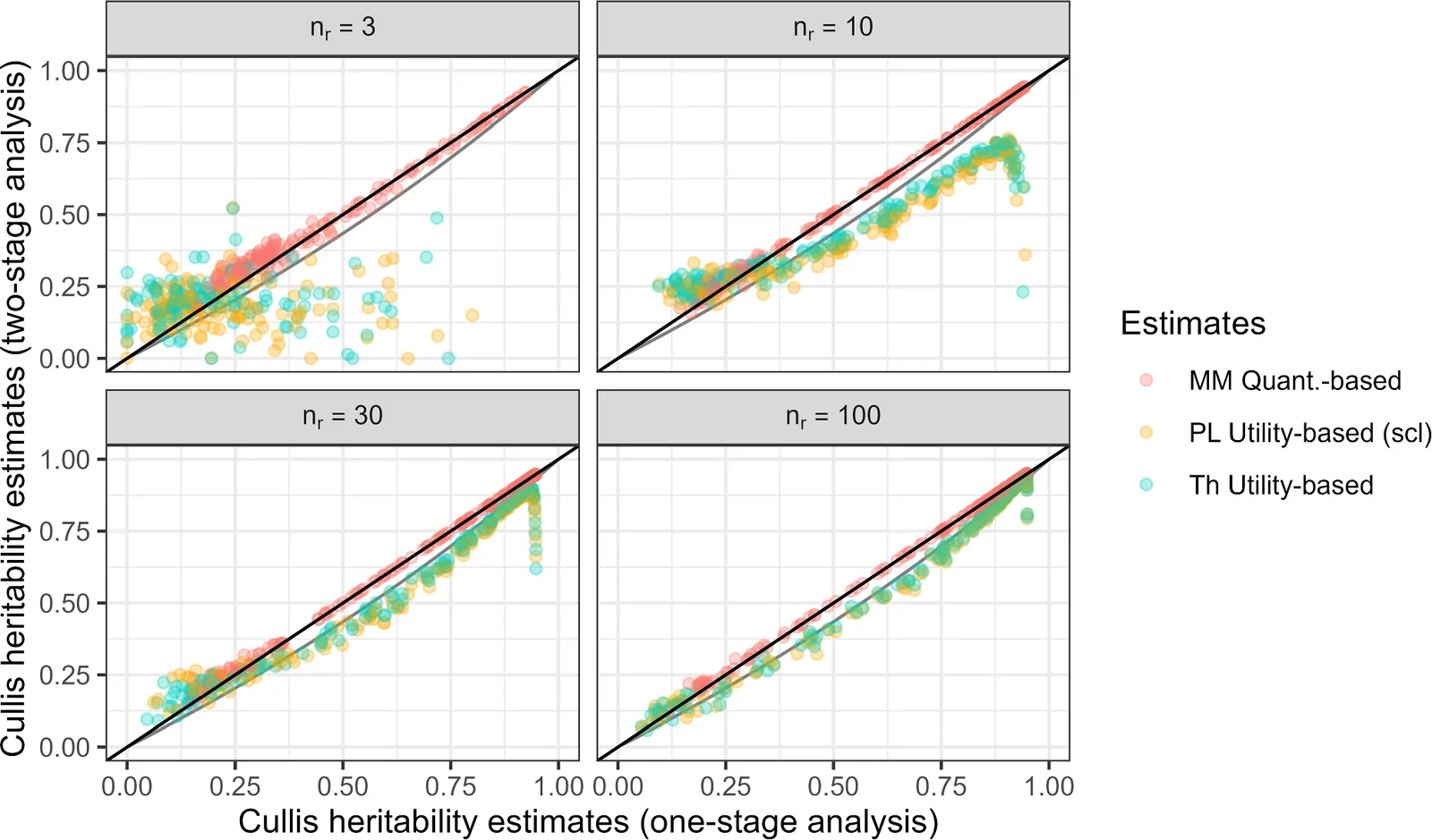
Reference versus estimated Cullis heritability for simulated grain yield in maize. Reference values were calculated using a one-step analysis of yield ($t\,\textrm{ha}^{-1}$), whereas estimated values were obtained using a two-step approach. The top panels correspond to the four values of the number of replicates ($n_r$). The x- and y-axis values represent the means of the estimates calculated over 30 simulation replicates with the same parameter settings (the disaggregated version of this figure is shown in Fig. S6). Simulated trials with heritability estimates ≤0 were excluded. Th, PL, and MM refer to estimates obtained using the Thurstonian, Plackett-Luce, and linear mixed-effects models, respectively. The dashed black line represents the 1:1 reference line, whereas the solid line represents the expected relationship for utility-based heritability

Figure 8 shows an overestimation for reference values of heritability lower than 0.25, affecting both estimates with quantitative data and utility-based estimates. Moreover, we observe that 3 replicates are insufficient to obtain reliable utility-based estimates. When the number of replicates are 10 or more, the relationship between estimated and reference heritability values becomes linear and close to one-to-one for quantitative-based estimates, whereas it follows an exponential pattern for utility-based estimates. The expected utility-based heritability (solid line) is calculated from the entry-mean broad-sense heritability, adjusted to account for the variability in ranking data as ($\hat{\sigma }_g^{2} / (\sigma _g^{2} + 1.3\,\sigma _{\varepsilon }^{2}/n_r)$), where the factor 1.3 adjusts for the difference in precision between utility- and quantitative- based measurements (Fig. S3). Notably, utility-based estimates of heritability are generally lower than quantitative-based estimates, which is expected given the greater uncertainty associated with rankings. The gap between quantitative- and utility-based estimates decreases as the number of replicates increases, since the high uncertainty inherent to ranking comparisons is compensated for by a larger sample size.

### Observed data example

Table 3 shows that genetic correlations in all four cases were high and close to 1, indicating that both quantitative yield evaluations and yield rankings reflect the same underlying genetic effects. For groundnut and early-maturity maize, genetic standard deviation estimates were relatively similar, as expected from our simulation analysis. In contrast, for sweetpotato, differences among genotypes were more evident when using quantitative data than when using ranking data, resulting in a higher estimate for genetic standard deviation of the former.

Heritability estimates for groundnut, early-maturity maize, and sweetpotato were consistently higher when derived from quantitative data ($\hat{\bar{H}}_{qt}^{2}$), reflecting the expected loss of information when using rankings ($\hat{\bar{H}}_{ut}^{2}$). Furthermore, heritability estimates based on farmers’ rankings ($\hat{\bar{H}}_{ut}^{2}$) were nearly identical to those obtained from rankings derived directly from quantitative yield data ($\hat{\bar{H}}_{ut_2}^{2}$), suggesting that farmers’ perceptions or ranking skills did not bias these heritability estimates. The most distinct result was observed for intermediate-maturity maize, where the genetic standard deviation estimated from quantitative data was too low, leading to a very low heritability estimate ($\hat{\bar{H}}_{qt}^{2}$), whereas the ranking-based analysis yielded higher genetic standard deviation and positive heritability estimates.

**Table 3:** Estimates of genetic parameters from quantitative and rank-based data for the observed yield in $t\,\textrm{ha}^{-1}$

Crop	$\hat{r}_{g^{qt},g^{ut}}$	$\hat{\sigma }_{g^{qt(\textrm{SC})}}$	$\hat{\sigma }_{g^{ut}}$	$\hat{\bar{H}}_{qt}^{2}$	$\hat{\bar{H}}_{ut}^{2}$	$\hat{\bar{H}}_{ut_2}^{2}$
Groundnut	0.961	0.179	0.161	0.359	0.229	0.218
Maize (Early-maturity)	0.997	0.204	0.205	0.607	0.525	0.581
Maize (Intermediate-maturity)	0.981	0.033	0.170	0.001	0.407	0.297
Sweetpotato	0.978	0.201	0.145	0.324	0.136	0.147

$\hat{r}_{g^{qt},g^{ut}}$ denotes the genetic correlation between genotypic effects estimated from quantitative and rank-based data. $\hat{\sigma }_{g^{qt(\textrm{SC})}}$ and $\hat{\bar{H}}_{qt}^{2}$ represent the genetic standard deviation (expressed in units of $\hat{\sigma }_{\textrm{res}}$) and Cullis heritability, respectively, estimated from yield measured in $t\,\textrm{ha}^{-1}$. $\hat{\sigma }_{g^{ut}}$ and $\hat{\bar{H}}_{ut}^{2}$ denote the genetic standard deviation and Cullis heritability estimated from observed rankings. $\hat{\bar{H}}_{ut_2}^{2}$ refers to the Cullis heritability estimated from rankings derived from measured yield ($t\,\textrm{ha}^{-1}$). All estimates obtained from ranking data correspond to utility-based estimates derived from the Thurstonian model

## Discussion

Ranking comparisons are a practical and reliable way for small-scale farmers to evaluate genotypes on farm. However, analytical methods for this type of data remain limited in the field of plant breeding. In this study, we develop and present a novel procedure that plant breeders can use for quantitative genetic analysis using ranking data and that provides a foundation for incorporating such data into subsequent multi-trial analyses. By using simulated and observed data, we demonstrated that our method can generate reliable and robust estimates of genotypic means, their variances, genetic standard deviation, and heritability. These estimates remain accurate as long as the number of replicates (generally around 30 replicates) used in the trial compensates for the uncertainty caused by a high ratio of genetic to residual standard deviation. These conditions are reasonable and feasible to achieve in on-farm testing, where this ratio is expected to be low.

This study provides the first methodological framework for integrating ranking data into the conventional two-stage quantitative genetic analysis widely used in breeding programs. The novelty lies not only in adapting the two-stage approach to ranking data, but also in establishing key methodological components, including the selection of appropriate models for ranking data analysis, weighting strategies for the second stage, and reliable estimation of heritability. In addition, we address several topics that remain largely unexplored in quantitative genetic studies using ranking data, including the comparison of Plackett-Luce and Thurstonian models, the evaluation of methodological limitations, and applications to decentralized on-farm breeding trials.

With our procedure, ranking evaluations for both quantitative and non-quantitative traits collected on-farm can be processed and analyzed in a similar way to conventional quantitative traits, offering a straightforward, efficient, flexible, and easy-to-implement approach. Moreover, this procedure can be adapted and extended to multi-trial analyses following the two-step approach developed for quantitative trait analyses in plant breeding (Smith et al. 2001; Möhring and Piepho 2009; Verbyla 2023). Its combination of adaptability to a quantitative genetic framework and user-friendly implementation in analytical tools could encourage the plant breeding research community to adopt it in their pipelines.

Based on our simulation study, we corroborate that reliable estimates of genotypic means and their standard errors can be obtained from ranking-data analyses. This is feasible when rankings are assumed to be linked to continuous underlying utilities, which is a property inherent to both the Thurstonian and Plackett-Luce models. The accuracy of these estimates depends on the genetic-to-residual standard deviation ratio and on the number of replicates per genotype. In most cases, 30 replicates were sufficient; however, a larger number of replicates was required when this ratio exceeded 2. Assuming a normally distributed underlying trait, only the Thurstonian model yielded accurate estimates of genotypic utility means, whereas the Plackett-Luce model exhibited a systematic bias. Nevertheless, if the goal is only to determine the overall ranking (i.e., the order) of genotypes, both models perform similarly.

The parameter constraints applied to the Thurstonian model allow the utilities to be interpreted in units of the residual standard deviation of the trial. This property enables a direct correspondence between utility- and quantitative-based estimates for the same trait. Such comparability is particularly advantageous when integrating information from trials involving different evaluation types (i.e., rankings versus quantitative measures), as the residual standard deviation can often be derived from standard protocols or historical data. Although similar constraints are applied to the Plackett-Luce model, the resulting genotypic utility means do not scale directly to the residual standard deviation due to the systematic bias noted above. We demonstrated that this bias can be corrected through a scaling factor; however, this factor is not constant and varies according to the genetic-to-residual standard deviation ratio and the number of replicates (Fig. S1). When the ratio is below 1 and approximately 30 replicates per genotype are available, the expected scaling factor for the Plackett-Luce estimates is approximately 0.9. After this adjustment, both models perform similarly in estimating genotypic means and genetic standard deviation.

The assessment of model performance further revealed that, unlike the standard errors obtained from an analysis of quantitative traits under a balanced design, those from both the Thurstonian and Plackett-Luce models are not constant and follow an almost exponential pattern, as previously reported by Bürkner et al. (2019) and Frick (2023). Furthermore, the standard errors derived from ranking data were substantially larger than those obtained from metric data, reflecting the expected loss of information. Under typical on-farm conditions, approximately 30% more replicates would be required to achieve a precision equivalent to that obtained from quantitative measurements. While this may seem discouraging, it is essential to realize that rankings have distinct advantages in data collection from both economic and logistical perspectives since they are easier to collect and do not require specialized instruments.

Our results confirm that the second step of our approach can accurately estimate genetic standard deviations when a linear mixed-effects model is fitted to genotypic utility mean estimates using weights derived from their associated variances. This requires accurate estimation of both the genotypic utility means and their associated variances, which, as mentioned above, is feasible when the experiment includes a sufficient number of replicates. By obtaining precise estimates of genetic standard deviation, we validated that the non-constant heterogeneity observed in the genotypic utility mean estimates (See Fig. 3) is properly accounted for by using the inverse of the quasi-variances as weights in the mixed model.

We acknowledge that using the full variance-covariance matrix of the estimated genotypic utility means in the second-step model in Eq. (7), as proposed by Piepho et al. (2012b), could be more efficient and informative than using quasi-variances. However, the variance-covariance matrix of the utility means is derived from contrasts relative to a reference genotype constrained to zero, which may limit the information available for estimating genetic standard deviation and heritability. A potential way to address this limitation is through a contrast-based network meta-analysis (C-NMA), which can analyze treatment effects estimated relative to a common reference while exploiting the full covariance structure of the contrasts (White et al. 2019; Karahalios et al. 2022). In this study, we opted for quasi-variances because raw observations and complete variance-covariance matrices are rarely shared. Moreover, the tricot approach is typically implemented by decentralized teams involving multiple institutions that need to exchange and aggregate information efficiently, making quasi-variances a practical summary for data sharing across locations and organizations. Nevertheless, when centralized data, raw observations, or variance-covariance matrices of estimated genotypic utility means are available, a two-step procedure that incorporates C-NMA may provide a more effective alternative.

We demonstrate that our model can be used to estimate heritability following the method of Cullis et al. (2006). The resulting estimates are consistent with our expectations, as those derived from utilities (based on ranking data) were lower than those obtained from quantitative data, consistent with previous findings (Ricard and Legarra 2010). We interpret this as evidence that the use of evaluations based on ranking data entails a loss of information, leading to lower heritability estimates. Finally, the fact that reasonable heritability estimates from utilities were obtained using the method of Cullis et al. (2006) validates that the prediction error variances were adequately represented in our mixed model.

The analysis of observed data demonstrated consistency and agreement between yield evaluations using rankings provided by farmers and quantitative measurements taken by technicians or farmers. This was reflected in high genetic correlations, indicating that genetic performance and differences among genotypes were similarly expressed across both evaluation methods. Sweetpotato had the lowest number of replicates, and yield data in $t\,\textrm{ha}^{-1}$ were measured by technicians. Under these conditions, it is expected that differences among genotypes would be more clearly reflected in the estimation of genetic standard deviation in $t\,\textrm{ha}^{-1}$ than when using ranking data. Interestingly, yield ($t\,\textrm{ha}^{-1}$) was recorded by farmers for the early-maturity maize trial, and the large number of replicates likely increased the precision of the genetic standard deviation estimates obtained from ranking data, making the rank- and quantitative-based estimates comparable. The trial for intermediate-maturity maize showed the lowest quantitative estimates for genetic standard deviation and heritability. This could be explained by the greater exposure of these genotypes to late-season stresses, such as early cessation of rainfall or heat stress, compared with early-maturity genotypes. Because genetic variation was very low, we observed that estimates based on ranking data were overestimated, highlighting the limitation of Cullis heritability under these particular conditions, which will be discussed in the caveats paragraph below. To conclude, results from observed data demonstrate that quantitative genetic analysis based on ranking data is feasible. The rankings provided by farmers are reliable and consistent with quantitative measurements taken by technicians, effectively reflecting farmers’ preferences and needs. Thus, our procedure offers breeders a useful tool to analyze this type of information.

It is known that small-scale farmers can accurately measure quantitative traits, which makes a ranking approach complementary or, in some cases, unnecessary for evaluating genotypes (Paliwal and Jain 2020; Nabateregga et al. 2025). In practice, our procedure could be particularly useful in experiments that involve other stakeholders, such as consumers or traders, and focus on the evaluation of non-quantitative traits, including preferences for color, flavor, overall preference, or marketability. These traits are key for the adoption of new genotypes, especially for small-scale holders.

A caveat identified in our approach arises when certain genotypes are consistently ranked at either extreme (highest or lowest) across all observations. This situation leads to undefined estimates of genotypic utility means and inflated variances, thereby reducing the accuracy of genetic standard deviation and heritability estimates. Such cases mainly occur in trials with a high true genetic-to-residual standard-deviation ratio and a limited number of replicates. Because our approach is intended for on-farm testing, where genetic variability is expected to be less expressed under untested field conditions and experimental variability is higher, we anticipate this ratio to remain below 2. Under these circumstances and based on our simulation study, approximately 30 replicates per genotype are sufficient for reliable estimations. We do not recommend removing these “problematic” genotypes, as doing so could underestimate genetic standard deviation. A more appropriate solution is to increase the number of replicates per genotype or restrict the analysis to the ranking (order) of the genotypic utility means estimates. As demonstrated by the Kendall correlation coefficients (see Fig. 5), the ranking itself is not affected by this limitation.

We identified two limitations related to the study that should be considered: (1) Only yield was analyzed in both the simulated and observed datasets, as it is typically assumed to be continuous and normally distributed. In fact, most traits with commercial value exhibit continuous distributions and result from the joint segregation of multiple genes, which generally leads to an approximately normal distribution (Falconer 1989). We acknowledge that some traits may have unknown probabilistic distributions. In these cases, it is worth evaluating whether the Central Limit Theorem ensures normality of the estimated genotypic utility means; and (2) although we consider the Cullis et al. (2006) method to be the most effective approach for estimating heritability in the analysis of trials with ranking data, we are aware that it can be imprecise and may yield unstable or negative estimates when the ratio of genetic to residual standard deviation is extremely low, such as the case of intermediate-maturity maize. Other methods for estimating heritability could be explored in the future. In Fig. S7, we calculate broad-sense heritability using the median of the variances of the genotypic utility mean estimates.

This study provides a basis for the analysis of ranking data in plant breeding. We suggest that future work may focus on: (1) extending the proposed framework to explicitly account for genotype × environment, genotype × year, and genotype × environment × year interactions, where environments are defined as groups of farms with similar biophysical conditions. This would enable the joint analysis of multiple trials and facilitate the assessment of genotype performance across diverse environmental conditions; (2) estimating and validating additional parameters such as genetic gain and genetic correlation between environments and/or traits; (3) developing and testing a method for utility-based multi-trait analysis that enables the simultaneous evaluation of varying traits; (4) improving utility-based estimates by incorporating data of farms where evaluations using both rankings and quantitative data have been conducted; and (5) Assessing the performance of the estimators under designs involving more than three genotypes per evaluation, the inclusion of ties, and/or within-farm replication to separate genotypic × farm interaction effects from residual variance.

From a practical breeding perspective, our results highlight the potential of ranking data in breeding programs. Farmers’ preferences and on-farm evaluations can be readily captured through ranking data, and our validated framework enables breeders to analyze this information and make informed decisions aligned with farmers’ needs.

## Supplementary Information

Below is the link to the electronic supplementary material.

Supplementary file 1 (docx 2886 KB)

## Data Availability

The observed data for groundnut and sweetpotato used in this study are publicly available and can be accessed at: Global multi-crop agricultural trial data supported by citizen science, Zenodo [https://doi.org/10.5281/zenodo.17112492], and are released under the Creative Commons Attribution-ShareAlike 4.0 International License (CC BY-SA 4.0). The observed maize data, however, were subject to CIMMYT data governance procedures and, at the time of publication, were still being prepared for open access; these data were therefore available from the authors upon reasonable request. The R functions and simulation workflow used in this study are publicly available at: - Source code available from: [https://github.com/hdorado/tricot-ranking-analysis] - Archived software available from: [https://doi.org/10.5281/zenodo.17942919] - License: [MIT License] Supplementary Information includes two sections (1. Supplementary Material and Methods and 2. Supplementary Figures), which are available via the Zenodo repository [https://doi.org/10.5281/zenodo.17941054] and under the terms of the Creative Commons License (CC BY 4.0),
