# Supplementary material for: Estimating on-farm genotypic performance and variability using ranking data
Source: Theor Appl Genet. 2026 Aug 18;139(9):231. doi: 10.1007/s00122-026-05319-1 (PMC13486096; doi:10.1007/s00122-026-05319-1)
Supplement: Supplementary file 1 [file 122_2026_5319_MOESM1_ESM.docx]

**Supplementary Information for:**

**Estimating on-farm genotypic performance and variability using ranking data**

Hugo Dorado-Betancourt^1,2,4,*^, Fred van Eeuwijk^1^, Jacob van Etten^3^, Julian Ramirez-Villegas^2,4^, Kauê de Sousa^3,5^, Happy Daudi^6^, Michael Ndegwa^7^, Thiago Mendes^8^, and Joost van Heerwaarden^1,2,*^

1. Mathematical and Statistical Methods (Biometris), Wageningen University & Research, Wageningen, the Netherlands

2. Plant Production Systems, Wageningen University & Research, Wageningen, the Netherlands

3. Digital Inclusion, Bioversity International, Montpellier, France

4. Climate Action, International Center for Tropical Agriculture (CIAT), Cali, Colombia

5. Department of Agricultural Sciences, University of Inland Norway, Hamar, Norway

6. Dryland crops program, International Maize and Wheat Improvement Center (CIMMYT), Nairobi, Kenya

7. Sustainable Agrifood Systems (SAS), International Maize and Wheat Improvement Center (CIMMYT), Nairobi, Kenya

8. International Potato Center (CIP), Nairobi, Kenya

*Correspondence: Hugo Dorado-Betancourt, ORCID: [hugo.dorado@wur.org](mailto:h.a.dorado@cgiar.org), ORCID: <https://orcid.org/0000-0002-0103-7505>, and Joost van Heerwaarden, ORCID: <https://orcid.org/0000-0002-4959-3914> ;e-mail: [joost.vanheerwaarden@wur.nl](mailto:joost.vanheerwaarden@wur.nl);

## **1. Supplementary Material and Methods**

## **1.1 Parameters of the bivariate normal distribution used to calculate the probability of obtaining the ranking** $\boldsymbol{R}_{\boldsymbol{k}}\mathbf{:}\boldsymbol{geno}\mathbf{5>}\boldsymbol{geno}\mathbf{8>}\boldsymbol{geno}\mathbf{1}$ **under the Thurstonian model**

The vector of utilities for the ranking $R_{k}$, $y_{k}=(y_{5k},y_{8k},y_{1k})'$, is assumed to follow a multivariate normal distribution with mean vector $\mu_{y_{k}}=(\mu_{5},\mu_{8},\mu_{1})'$ and covariance matrix

$$\Sigma_{y_{k}}=\left[ \begin{matrix} \sigma_{55} & \sigma_{58} & \sigma_{51} \\ \sigma_{58} & \sigma_{88} & \sigma_{81} \\ \sigma_{51} & \sigma_{81} & \sigma_{11} \end{matrix} \right]$$

After defining $U_{1k}= y_{5k}-y_{8k}$, $U_{2k}= y_{8k}-y_{1k}$, the joint distribution $U_{k}=(U_{1k},U_{2k})' ,$ follows a bivariate normal distribution with mean vector $\mu_{U_{k}}=(\mu_{5}-\mu_{8},\mu_{8}-\mu_{1})$ and covariance matrix $\Sigma_{U_{k}}$.

$$\Sigma_{U_{k}}=\left[ \begin{matrix} \sigma_{55}+\sigma_{88}-{2\sigma}_{58} & \sigma_{58}-\sigma_{51}-\sigma_{88}+\sigma_{81} \\ \sigma_{58}-\sigma_{51}-\sigma_{88}+\sigma_{81} & \sigma_{88}+\sigma_{11}-{2\sigma}_{81} \end{matrix} \right]$$

Further details (Böckenholt, 1992).

## **1.2 Estimating log worth values of the Plackett-Luce model via log likelihood function for ranking events with two genotypes**

We consider the random variable $D=y_{2}-y_{1}$*,* representing the difference between the utilitie*s* $y_{1}$ and $y_{2}$ associated with the preferences for genotypes 1 and 2, respectively. According to the Plackett–Luce (PL) model*,* $D$ is logistically distributed $\mu_{D}=\mu_{2}-\mu_{1}$ and scale parameter $S$ (where$,S=\sqrt{2}$ under the conventional PL model, but we keep it general here)*.*

For ranking comparisons, where ties are not allowed and only two items are assessed, the possible outcomes are: $geno 1>geno 2$ or $geno 2>geno 1$. These outcomes are modeled using a cumulative logistic distribution function, as follows:

$$P\left( geno 1>geno 2 \right)=P\left( y_{1}>y_{2} \right)=P\left( -(y_{2}-y_{1})>0 \right)=P\left( D<0 \right)=1/{(1+e^{\mu_{D}/S})}$$

or

$$P\left( geno 2>geno 1 \right)=P\left( y_{2}>y_{1} \right)=P\left( y_{2}-y_{1}>0 \right)=P\left( -D<0 \right)=1/{(1+e^{-\mu_{D}/S})}$$

The likelihood function to estimate $\mu_{D}$ is given by:

$$L(\mu_{D})= \left( \frac{1}{1+e^{\mu_{D}/S}} \right)^{W_{12}}\left( \frac{1}{1+e^{-\mu_{D}/S}} \right)^{W_{21}}$$

Where, $W_{12}$ represent the number of times genotype 1 outperforms genotype 2, and $W_{21}$ represent the number of times genotype 2 outperforms genotype 1.

From the logarithm function of $L(\mu_{D})$ we obtain:

$$Log\left( L(\mu_{D}) \right)= -W_{12}\left[ \log\left( 1+e^{\mu_{D}/S} \right) \right]-W_{21}[\log\left( 1+e^{-\mu_{D}/S} \right)]$$

We optimized the system based on the first derivate of $Log\left( L(\mu_{D}) \right)$

$$\frac{\partial Log\left( L(\mu_{D}) \right)}{\partial\mu_{D}}=\frac{-W_{12}}{S} \left( \frac{e^{\frac{\mu_{D}}{S}}}{1+e^{\frac{\mu_{D}}{S}}} \right)+\frac{W_{21}}{S}\left( \frac{W_{21} e^{-\frac{\mu_{D}}{S}}}{1+e^{-\frac{\mu_{D}}{S}}} \right)=0$$

Operating the terms we simplified to

$$W_{21} e^{-\mu_{D}/S}-W_{12} e^{\mu_{D}/S} +\left( W_{21}-W_{12} \right)=0$$

Multiplying all the terms by $e^{\mu_{D}/S}$ and grouping them

$$W_{12} e^{{2\mu}_{D}/S} - e^{\mu_{D}/S} (W_{21}-W_{12})-W_{21} = 0$$

By substituting the variable $X=e^{\mu_{D}/S}$, the equation transforms into a quadratic system with two roots, of which only the positive root is feasible for the solution of $X$, yielding

$X=W_{21}/W_{12}$

Reversing the substitution

$$e^{\mu_{D}/S}=W_{21}/W_{12}$$

We can then derive the estimator for as $\mu_{D}$

$$\hat{\mu}_{D}=S log(W_{21}/W_{12})$$

Finally, by applying the constraint $\hat{\mu}_{1}=0$, we obtain that $\hat{\mu}_{D}$ is the estimation of the relative genotypic value of the genotype 2:

$$\hat{\mu}_{D}=\hat{\mu}_{2}-\hat{\mu}_{1}= \hat{\mu}_{2} =S log(W_{21}/W_{12})$$

## **1.3 Scaling constant for equivalence between estimated genotypic values using the Thurstonian and Plackett-Luce models**

The relationship of estimated utility means between Thurstonian and Plackett-Luce models can be expressed as $Thurstonian estimates\approx C* PL. estimates$, where $C$ is a scaling constant that makes both estimates approximately equivalent. For experiments with two genotypes, $C$ is approximately 0.8309, derived from the equivalence between the normal and logistic distributions However, as the number of genotypes increases, $C$ may depend on the genetic and residual variances, and the number of replicates. In trials where the genetic standard deviation is lower than the residual variance and the number of replicates exceeds 30, which are the most common conditions in tricot trials, the value of $C$ is close to 0.9. The extended version with details is shown below.

Ranking comparison of two genotypes

The scaling factor for the equivalence between the Thurstonian and Plackett-Luce estimates *C*, is derived by analyzing the probabilistic distribution of the difference $y_{1k}-y_{2k}$, where $y_{1k}$ and $y_{2k}$ represent underlaying utilities for genotypes 1 and 2, respectively, provided by a farmer *k*. The distribution of this difference depends on the assumptions underlying each model.

As mentioned in Section 2.2, the Thurstonian model assumes that $y_{jk}$ follows a normal distribution with location and scale parameters $\mu_{j}$ and $S=\sigma_{e}$, respectively. In contrast, the Plackett-Luce model assumes a Gumbel-distributed $y_{jk}$ with location parameter $\mu_{j}$ and scale parameter $S$. For identification purposes in estimation, we impose the constraints $\mu_{1}=0$ and $S=1$ for both models. Notice that the variance of a standard Gumbel distribution $(\pi^{2}/6$) is higher than that of the standard normal distribution (which is equal to 1). Given these assumptions and constraints, the difference $D_{j}=y_{2k}-y_{1k}$ follows a normal distribution with mean $\mu_{2}$ and standard deviation $\sqrt{2}$ under the Thurstonian model, whereas $D_{j}$ follows a logistic distribution with mean $\mu_{2}$ and scale parameter 1 under the Plackett-Luce model (Yellott, 1977).

The cumulative functions $\Phi$ and $\psi$, corresponding to the standard normal and standard logistic distributions, respectively, can be approximated using the scaling constant $k_{0}=1.702$, such that $\Phi\left( y \right)\approx\psi\left( k_{0}y \right)$ (Camilli, 1994). This constant has been used to relate logit and probit transformations in dichotomous variable analysis, where $logit(y)\approx1.702 \Phi^{-1}\left( y \right)$, (Baker & Kim, 2004; Savalei, 2006). The first derivative of $\psi\left( k_{0}y \right)$ corresponds with a logistic function with location parameter $\mu=0$ and scale parameter ${S=1/k}_{0}$ as is shown in Eq. A(1).

$$\psi^{'\left( k_{0}y \right)}=\frac{k_{0}\exp\left( -k_{0}y \right)}{\left( 1+\left( -k_{0}y \right) \right)^{2}} Eq. A(1)$$

Applying the same principle to a random variable for the differences of two utilities, $D$, we scale it by its respective standard deviation: $\Phi\left( D/\sqrt{2} \right)\approx\psi\left( 1.702*D/\sqrt{2} \right)$. This converts $\psi$ in a cumulative logistic distribution with a scale parameter $S=\sqrt{2}/1.702 \approx0.8309$.

In section 1.2 of Supplementary Material and Methods, we analyzed the effect of the scaling factor $S$ on the estimation of genotypic utilities means $\mu_{1}$ and$\mu_{2}$, obtaining the log-likelihood estimator corresponding to $\hat{\mu}_{2}=S log(W_{12}/W_{21})$, where, $W_{12}$ represents the number of times genotype 1 outperforms genotype 2, and $W_{21}$ represents the number of times genotype 2 outperforms genotype 1. From this result, we can deduce that, for the comparison of two genotypes, the scaling constant required to make Plackett–Luce–estimated genotypic values to Thurstonian–estimated values is approximately equivalent to $S=0.8309$.

Ranking comparison of more than two genotypes

When more than two genotypes are included in the experiment, defining a general scaling constant for converting estimated genotypic values from the Plackett-Luce model to the Thurstonian model becomes more complex. Such is the case of tricot data, where trials often involve more than ten genotypes, and ranking events are modeled either as a cumulative multivariate distribution under the Thurstonian model or as products of multinomial logistic functions under the Plackett-Luce model. We approximated a scaling constant value using our simulated data by performing a simple linear regression without an intercept between both estimates. The results are shown in Fig. S1 where we can see that in the simulated experiments where the genetic-to-residual standard deviation ratio was below 1 and the number of replicates exceeded 30, the scaling constant for estimates was approximately 0.9. In contrast, experiments that yielded unstable estimates, defined by a ratio of variation greater than 1 and fewer than 30 replicates, had a clear influence on the estimated scaling constant.

## **1.4. Estimation of genetic correlation between two ranked-traits using a multi-trait model.**

We estimate genetic correlations involving at least one ranked trait by extending model (3) into a multi-trait model with two traits:

$$\left[ \begin{matrix} \hat{\mu}^{t1} \\ \hat{\mu}^{t2} \end{matrix} \right]=\left[ \begin{matrix} X^{t1} & 0 \\ 0 & X^{t2} \end{matrix} \right] \left[ \begin{matrix} \beta^{t1} \\ \beta^{t2} \end{matrix} \right]+\left[ \begin{matrix} Z^{t1} & 0 \\ 0 & Z^{t2} \end{matrix} \right]\left[ \begin{matrix} u^{t1} \\ u^{t2} \end{matrix} \right]+\left[ \begin{matrix} \varepsilon^{t1} \\ \varepsilon^{t2} \end{matrix} \right] (5)$$

Here, superscripts t1 and t2 denote trait 1 and trait 2, respectively. Where, $\hat{\mu}^{t1}$ and $\hat{\mu}^{t2}$ are vectors of estimated genotypic means for traits 1 and 2 respectively, all terms retain the same interpretation as in model (3) and the same distinction for the two traits in superscripts, $\beta$ and $u$ are vectors of fixed and random effects, with design matrices, $X$ and $Z$ respectively, and $\varepsilon$ is the residual vector.

The composed random vectors $[u^{t1}$, $u^{t2}]$ and [$\varepsilon^{t1}$, $\varepsilon^{t2}]$ are assumed to follow multivariate normal distributions with mean zero and covariance structures $G\otimes I$ and $R\otimes I$ respectively, where $\otimes$ is the Kronecker product. The genetic covariance matrix G is unstructured, with diagonal elements $\sigma_{g^{t1}}^{2}$ and $\sigma_{g^{t2}}^{2}$, and off-diagonal elements $\sigma_{g^{t1}\times g^{t2}}$, representing the genetic covariance between traits. Similarly, $R$ contains the residual variances $\sigma_{\varepsilon^{t1}}^{2}$ and $\sigma_{\varepsilon^{t2}}^{2}$ and residual covariance is $\sigma_{\varepsilon^{t1}\times\varepsilon^{t2}}$. Diagonal matrices containing the inverses of the quasi-variances of the genotypic mean estimates for each trait are used as weights in fitting model (5).

The genetic correlation is calculated according to:

$$r_{g^{t1},g^{t2}}=\frac{\sigma_{g^{t1}\times g^{t2}}}{\sigma_{g^{t1}}*\sigma_{g^{t2}}} (6)$$

This concept of genetic correlation applies to either two traits evaluated using ranking data or one trait evaluated with ranking data and another with quantitative data.

For true standard deviations lower than 1, the number of replicates per genotype tends to stabilize around 1.30.

## **2. Supplementary Figures**

**Fig. S1** Effect of the number of replicates ($n_{r}$) and true genetic standard deviation $(\sigma_{geno})$ on the scaling factor for equivalence between Thurstonian (TH) and Plackett-Luce (PL) estimates. The scaling factor is the regression slope of Thurstonian on Plackett–Luce estimates

**Fig. S2** True versus estimated genetic standard deviation (SD) for simulated maize grain yield across different number of replicates. The top panels correspond to weights used to fit the mixed-effect model that derived the estimates (inverse of genotypic means variances in the left, inverse of quasi-variances in the right). The y-axis values represent the means of the estimates obtained over 30 simulation replicates with the same parameter settings. Both axes are given in standardized units of $\sigma_{e}$. Th, PL, and MM refer to estimates obtained using the Thurstonian, Plackett–Luce, and linear mixed-effects models, respectively. Utility-based estimates from the Plackett–Luce model were scaled to match the units of the Thurstonian estimates. The solid black line represents the 1:1 line. Vertical dashed lines highlight the results of a trial with $\sigma_{g}=0.8$ and $\sigma_{e}=0.9$. The number of replicates ($n_{r}$) is 30

**Fig. S3** Effect of the number of replicates ($n_{r}$) and genetic standard deviation $(\sigma_{geno})$ on the ratio of -standard-error estimates for genotypic-means estimates: ranking vs quantitative data analysis. The top panels models for ranking data Thurstonian and Plackett-Luce.

**Fig. S4** Defined and undefined solutions of the log-likelihood function for the Thurstonian and Plackett-Luce models when comparing two genotypes: Case I refers to simulations where both genotypes are preferred at least once; Case II, where only one genotype is consistently preferred

**Fig. S5**. True versus estimated genetic standard deviation (SD) for simulated maize grain yield across different number of replicates. The top panels correspond to the four possible values for the number of replicates ($n_{r}$). The y-axis values represent the estimates obtained of 30 simulation replicates with the same parameter settings. Both axes are given in standardized units of $\sigma_{e}$. Th, PL, and MM refer to estimates obtained using the Thurstonian, Plackett–Luce, and linear mixed-effects models, respectively. Utility-based estimates from the Plackett–Luce model were scaled to match the units of the Thurstonian estimates. The solid black line represents the 1:1 line. Vertical dashed lines highlight the results of a trial with $\sigma_{g}=0.8$ and $\sigma_{e}=0.9$

**Fig. S6** Reference versus estimated heritability for simulated maize grain yield across different numbers of replicates. Reference values are calculated using a one-step analysis on yield (in tons/ha), while estimated values are calculated using a two-step approach. The top panels correspond to the four possible values for the number of replicates ($n_{r}$). The x- and y-axis values represent the estimates obtained of the 30 simulation replicates with the same parameter settings; simulated trials with heritability estimates ≤ 0 were discarded. Th, PL, and MM refer to estimates obtained using the Thurstonian, Plackett–Luce, and linear mixed-effects models, respectively. The dashed black line represents the 1:1 reference line. The solid line is the expected values for utility-based heritability.

**Fig. S7** Reference versus estimated entry-mean heritability for simulated maize grain yield across different numbers of replicates. Reference values were calculated from the analysis of yield (tons/ha) using the estimated residual variance, while estimated values were obtained by using the median of the variance of genotypic mean estimates, divided by two, to approximate the residual variance. The top panels correspond to the four possible values for the number of replicates ($n_{r}$). The x- and y-axis values represent the means of the estimates obtained over 30 simulation replicates with the same parameter settings. Th, PL, and MM refer to estimates obtained using the Thurstonian, Plackett–Luce, and linear mixed-effects models, respectively. The dashed black line represents the 1:1 reference line. The solid line is the expected values for utility-based heritability.
